# Structural Transition-Induced Raman Enhancement in Bioinspired Diphenylalanine Peptide Nanotubes

**DOI:** 10.1021/acsami.1c22770

**Published:** 2022-03-07

**Authors:** Sawsan Almohammed, Agata Fularz, Mohammed Benali Kanoun, Souraya Goumri-Said, Abdullah Aljaafari, Brian J. Rodriguez, James H. Rice

**Affiliations:** † School of Physics, University College Dublin, Belfield, Dublin D04 V1W8, Ireland; ‡ Conway Institute of Biomolecular and Biomedical Research, University College, Dublin, Belfield, Dublin D04 V1W8, Ireland; § Department of Physics, College of Science, King Faisal University, P.O. Box 400, Al-Ahsa 31982, Saudi Arabia; ∥ Physics Department, College of Science and General Studies, Alfaisal University, P.O. Box 50927, Riyadh 11533, Saudi Arabia

**Keywords:** semiconducting materials, FFNTs, Raman scattering, surface-enhanced Raman spectroscopy, HOMO, LUMO

## Abstract

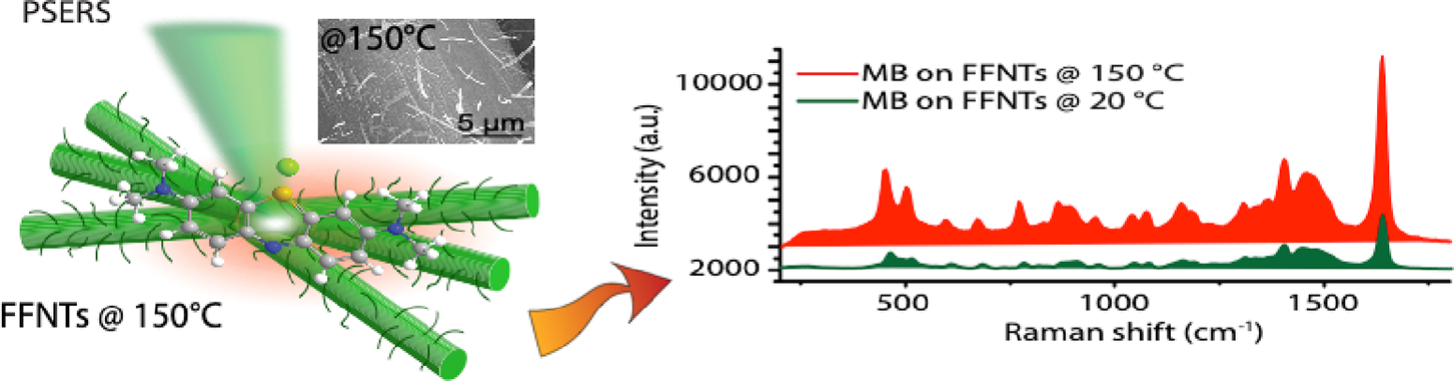

Semiconducting materials are increasingly proposed as alternatives to noble metal nanomaterials to enhance Raman scattering. We demonstrate that bioinspired semiconducting diphenylalanine peptide nanotubes annealed through a reported structural transition can support Raman detection of 10^–7^ M concentrations for a range of molecules including mononucleotides. The enhancement is attributed to the introduction of electronic states below the conduction band that facilitate charge transfer to the analyte molecule. These results show that organic semiconductor-based materials can serve as platforms for enhanced Raman scattering for chemical sensing. As the sensor is metal-free, the enhancement is achieved without the introduction of electromagnetic surface-enhanced Raman spectroscopy.

## Introduction

Surface-enhanced Raman spectroscopy (SERS) is a powerful vibrational technique that allows for highly sensitive structural detection of low-concentration analyte molecules.^1,2^ The SERS signal is dependent on the materials used to fabricate the substrate, and in the past few years, semiconductor-based SERS substrates have gained significant research interest as a means to eliminate the cost associated with noble metal nanoparticles and to exploit the chemical enhancement mechanism to improve the SERS signal through, for example, charge transfer-related processes.^3−10^

Several strategies have been developed and explored to optimize the performance of semiconductor-based SERS substrates, including structural phase transitions and defect engineering that can enhance the charge transfer resonance effect.^9−14^ Phase transitions of metal oxides and dichalcogenides, including vanadium dioxide nanosheets^13^ and MoX_2_,^14^ have been reported to enhance Raman scattering. In the case of MoX_2_, the enhancement has been ascribed to the highly efficient charge transfer from the Fermi energy level of 1T-MoX_2_ to the highest occupied molecular orbital (HOMO) of the probe molecule.^14^ Similarly, plasma-induced defect formation and band gap shift in zinc oxide and the coupling of energy levels between zinc oxide and probe molecules result in an enhanced SERS signal.^10^ These substrates reported an enhancement factor as high as 10^5^ with a detection limit of ∼10^–6^ M, which are higher than that for zinc oxide before plasma treatment but still lower than those reported for metal-based SERS substrates (typically 10^–9^ M).^10^

Bioinspired semiconducting diphenylalanine peptide nanotubes (FFNTs) have attracted significant interest as a SERS substrate due to their biocompatibility and piezoelectric and pyroelectric properties, as well as their thermal and chemical stability and wide band gap.^15−17^ Studies have shown that the thermally induced structural transition of FFNTs leads to profound morphological changes from hollow hexagonal FFNTs to closed fiber-like structures at temperatures between 140 and 200 °C.^18^ During the process, there is a reconstruction of the covalent bonds, causing the amine and carboxyl groups to irreversibly form circular chains and release water.^19^ These morphological changes also result in extensive modification of the electronic properties of the native structures, resulting in new electronic states 1.8 eV below the conduction band (CB).^20^ Thermal annealing of the FFNTs leads to a new phase of refolded antiparallel β-sheet nanostructures with a broad network of hydrogen bonds, resulting in extensive modification of the electronic properties of the native structures.^20^ The introduction of the electronic states following thermal annealing can potentially affect charge transfer processes and thereby chemical enhancement of Raman intensity. Here, we show for the first time that the annealing-induced FFNT structural transition results in SERS enhancement. The ∼8-fold increase observed compared to pristine FFNTs is attributed to the enhanced charge transfer from thermally induced electronic states below the CB. A detection limit as low as 10^–7^ M was achieved for various dye and DNA-based molecules, which is higher than the detection limit (10^–4^ M) of pristine FFNTs, and a step closer to realizing the detection limits of SERS-based substrates but with bioinspired materials. These findings open up the possibility of using peptide-based materials for applications where charge transfer is essential to improve the sensitivity of detection such as a chemical sensor for biomedical applications. These results also show that organic semiconductor-based materials can serve as highly efficient platforms for enhanced Raman scattering for chemical sensing. As the sensor is metal-free, the enhancement is achieved without electromagnetic SERS.

## Results

**Figure 1:**
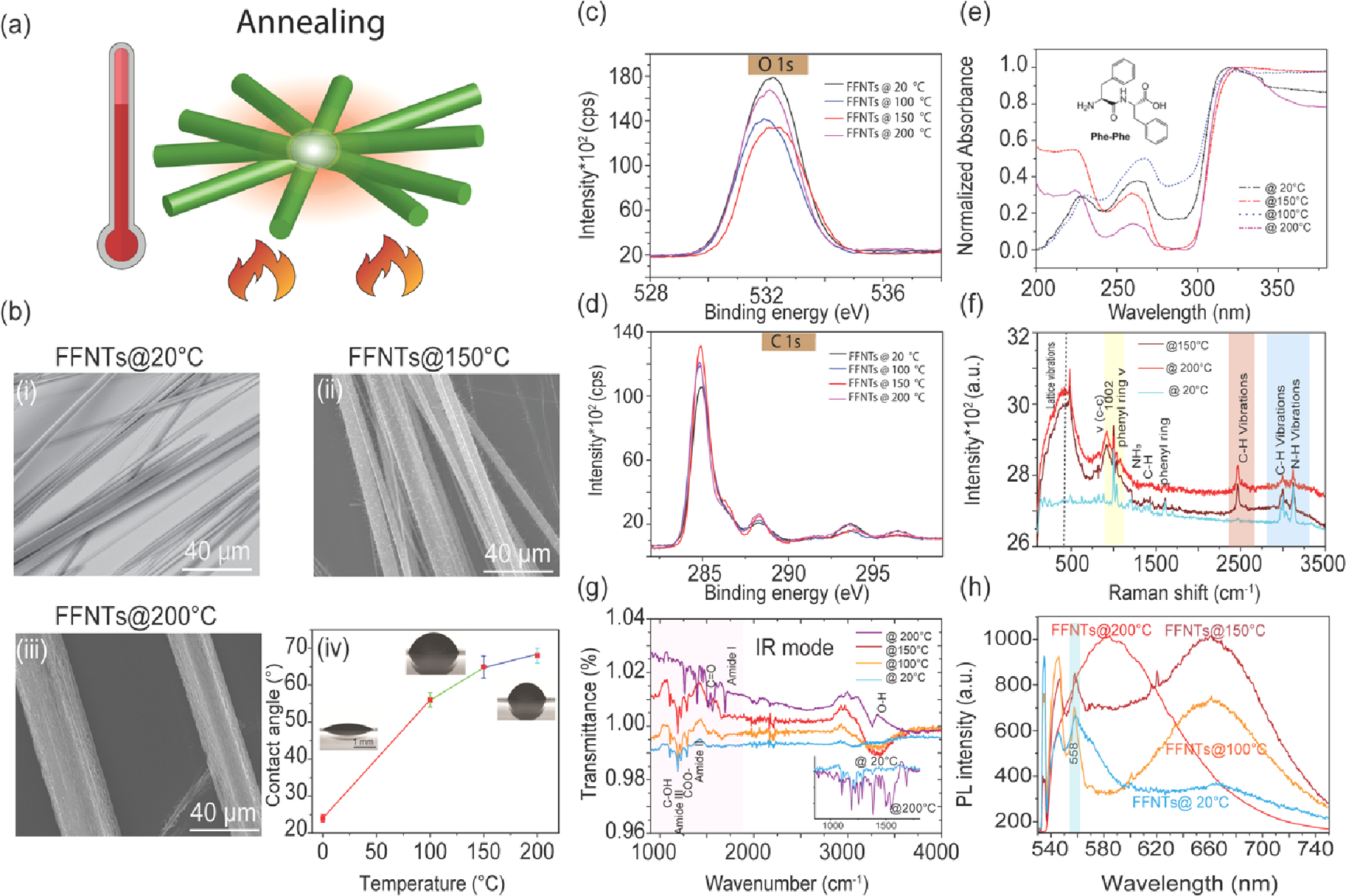
(a) Schematic showing the annealing of FFNTs. (b) SEM images of FFNTs annealed at (i) 20, (ii) 150, and (iii) 200 °C and (iv) the corresponding contact angles. (c,d) XPS spectra of the FFNTs before and after annealing. (e) UV–vis absorption spectra of the FFNTs as a function of temperature. The inset in (e) is the chemical structure of FF. (f) Raman measurements of the FFNTs at different temperatures. (g) FTIR measurements of the FFNTs as a function of temperature. The inset is the transmittance of the FFNTs at 20 °C and 200 °C. (h) PL emission spectra (532 nm excitation wavelength) from pristine FFNTs and FFNTs annealed at different temperatures. SEM, XPS, UV–vis, Raman, FTIR, and PL measurements of annealed FFNTs were performed without probe molecules.

The process to heat (at temperatures of 100, 150, and 200 °C) FFNTs prepared using a concentration of 2 mg/mL that was drop-cast on glass coverslips and dried at room temperature is illustrated in Figure 1a. The pristine and annealed FFNTs were characterized using scanning electron microscopy (SEM) (Figures 1b1–iii and S1). As the temperature increased from room temperature to 150 °C, the FFNTs became fibers (Figure 1bii) with a diameter of ∼214 ± 56 nm (*n* = 20). Upon heating to 200 °C, the fiber diameter became ∼175 ± 50 nm (*n* = 20) (Figure 1biii). The morphology change observed in SEM as the temperature increased to 200 °C is consistent with morphological changes associated with the irreversible structural transition from a noncentrosymmetric hexagonal space group to a centrosymmetric orthorhombic space group reported in the literature.^20,21^ Contact angle measurements (Figure 1biv) show that as the temperature increased from 20 to 200 °C, the surface of the FFNTs became less hydrophilic with the contact angle increasing from 25° (at 20 °C) to 67° (at 200 °C).

To corroborate this phase transformation, we measured the atomic mass of carbon and oxygen in the FFNTs before and after the thermal treatment using energy-dispersive X-ray spectroscopy (EDX). As evidenced by EDX mapping (Figure S2), both the mass concentration and atomic weight of O decreased as the temperature increased. In contrast, the weight percentage of C increased with temperature, consistent with the loss of water.^20,21^ In confirmation, the weight of the fibers decreased from 0.22 g at 20 °C to 0.18 g at 200 °C.

Atomic and mass concentrations of C and O before and after the thermal treatment were also observed to decrease as the temperature exceeded 150 °C using X-ray photoelectron spectroscopy (XPS) (Figures 1c,d, S3, and S4), consistent with a structural transition and loss of water.^20,21^ The energies of 284.8, 286.9, and 290.2 eV are assigned to C–C, C–N or C=O, and O–C=O bonds, respectively. The lowest energy peak of 282.4 eV is attributed to environmental contamination. The O 1s XPS spectrum is located at 532 eV and attributed to C=O or lattice oxygen. The largest changes in FFNTs upon heating are in the binding energies of the amide bond (including the carbonyl bond) and the amine group.^22^

At room temperature, FFNTs form a noncentrosymmetric hexagonal (P61) structure. Such hexagonal-shaped FFNTs consist of hollow tubular nanochannels formed by six stacked FF molecules that interact with each other by strong and weak hydrogen bonds and aromatic interactions. Water molecules fill the nanochannels, and each linear FF (H_2_ N–Phe–Phe–COOH) molecule has two hydrophobic aromatic rings in the side chains between the amino and the carboxylic ends.^19^ The hydrogen bonding motif for FFNTs is two −NH_3_ −COO– head-to-tail chains in a two-dimensional sheet.^19−26^ When the temperature increases over 150 °C, the hexagonal FF is transferred into an orthorhombic (*P*2_1_2_1_2) cyclo-FF crystalline structure.^19−26^ This structure is an irreversible reconstruction of the chemical covalent bonds and the production of cyclic peptides from the linear FF molecules. As a result of this cyclization process, both amino and carboxyl groups are connected to each other by strong covalent bonds, leading to the formation of a circular chain. This cyclization process is followed by the release of water.^19^

Optical absorption spectroscopy provides a direct measurement of the electron energy spectrum (Figure 1e). FFNTs are well known to have an absorption band located at 220 nm (5.6 eV), indicative of a π–π* electronic transition of the phenylalanine ring, with subpeaks located at 265 nm (4.68 eV) and 253 nm (4.90 eV), due to contributions from aromatic residues in FF.^20,21,23,24^ Following heating from 100 to 200 °C, these UV peaks maintain their location, indicating that the spectral region at ∼260 nm is not affected by the structural phase transition, consistent with literature results.^20,21,23,24^ A second FFNT absorption band at 340 nm red-shifts to 345 nm after annealing at 150 °C, which is associated with the molecular transformation of linear FF to cyclic FF molecules. In addition, the band gap of FFNTs, as calculated from optical absorption measurements, decreases from around 4.4 eV at 20 °C to 3.86 eV at 150 °C (Figure S5). The red shift along with changes in the FFNT band gap potentially arises from changes in the hydrogen bonding, which promotes the formation and construction of a new secondary β-sheet structure, as reported in the literature.^20,21,23,24^ It can be assumed that optically excited electronic transitions at 345 nm occur from newly created electron energy levels related to the specific secondary organizations. This new electronic structure appears as a result of the formation of intermolecular hydrogen bonds of β-sheet structures.^20,21,23,24^

Raman spectroscopy can provide unique optical signatures of materials due to the high sensitivity of specific vibrational modes of the chemical bonds in a molecule. For this reason, Raman measurements of FFNTs were undertaken. Figure 1f illustrates Raman spectra of the FFNTs, which correspond to the vibrations and chemical structural elements of FFNTs.^25,26^ As the temperature increases from 100 to 200 °C, all Raman regions of FFNTs change and slightly increase in intensity. The most significant region influenced by temperature (Figure 1f) is the region between 2800 and 3350 cm^–1^. It is reasonable to suggest that water molecules affected the bands located at 2920, 2938, and 2966 cm^–1^ assigned to C–H stretching vibrations. Hence, water evaporation observed at a high temperature of ∼150–200 °C leads to the frequency shift of these vibrations^25^ that results in significant changes in the crystal lattice. Interestingly, at both 150 and 200 °C, a new band located at 2500 cm^–1^ (C–H vibration) starts to grow, which, according to the literature, is associated with a structural transition of FFNTs.^20,27,28^ Studies have shown that the structure and the features of the FFNTs are strongly influenced by the water content in the subsystem. Water molecules are weakly bound with FFNTs and can move along the nanochannel. Introducing heat into the system results in water evaporation^29^ and cyclization of FF molecules.^30−32^ Annealed FFNTs remain stable at high laser power up to 100 mW (Figure S6).

Fourier transform infrared spectroscopy (FTIR) is a standard method for monitoring the chemical composition of organic and inorganic materials and the conformation kinetics of bioinspired and biological materials. FTIR has been used to identify secondary structural changes such as the detection of β-sheet structures that accompany the formation of fibrils.^20^ For this reason, we have characterized the chemical structure of FFNTs before and after heating using FTIR, as shown in Figure 1g.

Starting from 20 °C, the wavenumbers of 1600–1700 cm^–1^ relate to the amide I vibrational band signature of the secondary peptide arrangement. The amide I band at 1633 is red-shifted at 150 °C to ∼1650 cm^–1^ [C=O stretching (amide I)]. Studies have shown that a red shift and an increase in the intensity of amide I bands are strong indications of the formation of more hydrogen bonds and β-sheet rearrangement. More intense bands started to appear as a function of heating at 150 °C, such as the band at 1536 cm^–1^, which is associated with N–H bending (amide II band). The significant increase in intensity of this band is strong evidence of increasing hydrogen bonding. Other amide II regions were observed at 1693 and 1746 cm^–1^. Bands at 1746 cm^–1^ that started to appear with increasing temperature are attributed to C=O stretching. At 200 °C, the peaks corresponding to antiparallel β-sheets are still observed, but their intensity is weaker. Additional peaks are observed in the amide II region at 1693 and 1744 cm^–1^, which could be an indication of a structural phase transition.^21−25^ The shoulder at 3223 cm^–1^ is assigned to N–H in-plane vibrations. The red shift and split of the N–H stretching band at 3223 cm^–1^ and 3354 cm^–1^ after heating to 150 °C are evidence of the formation of additional hydrogen bonds with the participation of the amide N–H group.^20−30^ Secondary structures can be observed at wavenumbers related to the amide I vibrational band between 1600 and 1700 cm^–1^. The new fibre phase has a significantly different FTIR frequency structure, which indicates refolding of the original peptide secondary structures, in line with literature reports.^30−32^

In order to examine the secondary structure of the FFNT ensembles in their native and thermally induced phases, we applied circular dichroism (CD) (Figure S7). For the native phase, the CD spectrum of FFNT (Figure S7) exhibits a positive band with two broad peaks of ellipticity as a function of temperature. The first maximum in ellipticity is Δε ≈ 70 mdeg at ∼228 nm, indicative of an *n*–π* transition.^20^ The lower maxima in ellipticity at ∼210 nm (Δε ≈ 10 mdeg) and 203 nm result from *n*–π* and π–π* transitions (energy transition in the amide group), respectively. As the temperature increased, the sign of the peak’s intensity changed and gradually decreased, and the peak became broad. At around 150–200 °C, the peak became negative. Such a change in CD reflects a major structural rearrangement of FFNT ensembles and the creation of a new secondary structure, in line with literature reports.^20^

The photoluminescence (PL) spectra were recorded in both the pristine and annealed phases. In the pristine phase (Figure 1h), when the aromatic FFNTs are excited at ∼532 nm, fluorescence emission peaks at ∼553, 546, and 557 nm were observed, which are well-known PL signatures of phenylalanine residues.^20^ The second PL peak appears at ∼660 nm only after the structural transition of FFNTs. This band was blue-shifted to around 585 nm at 200 °C, which could be an indication of the β-sheet arrangement stabilizing the network of hydrogen bonds between the polypeptides. According to the literature, the reconstructive structural transition leads to a new phase of refolded antiparallel β-sheet nanostructures with a broad network of hydrogen bonds, resulting in extensive modification of the electronic properties of the native structures.^20^ This visible PL effect, happening as a result of the photon excitation of low-energy electronic transitions, is attributed to the intrinsic electronic structure of the newly assembled β-sheet arrangement.^20^

### Peptide Semiconductor-Enhanced Raman Scattering

**Figure 2:**
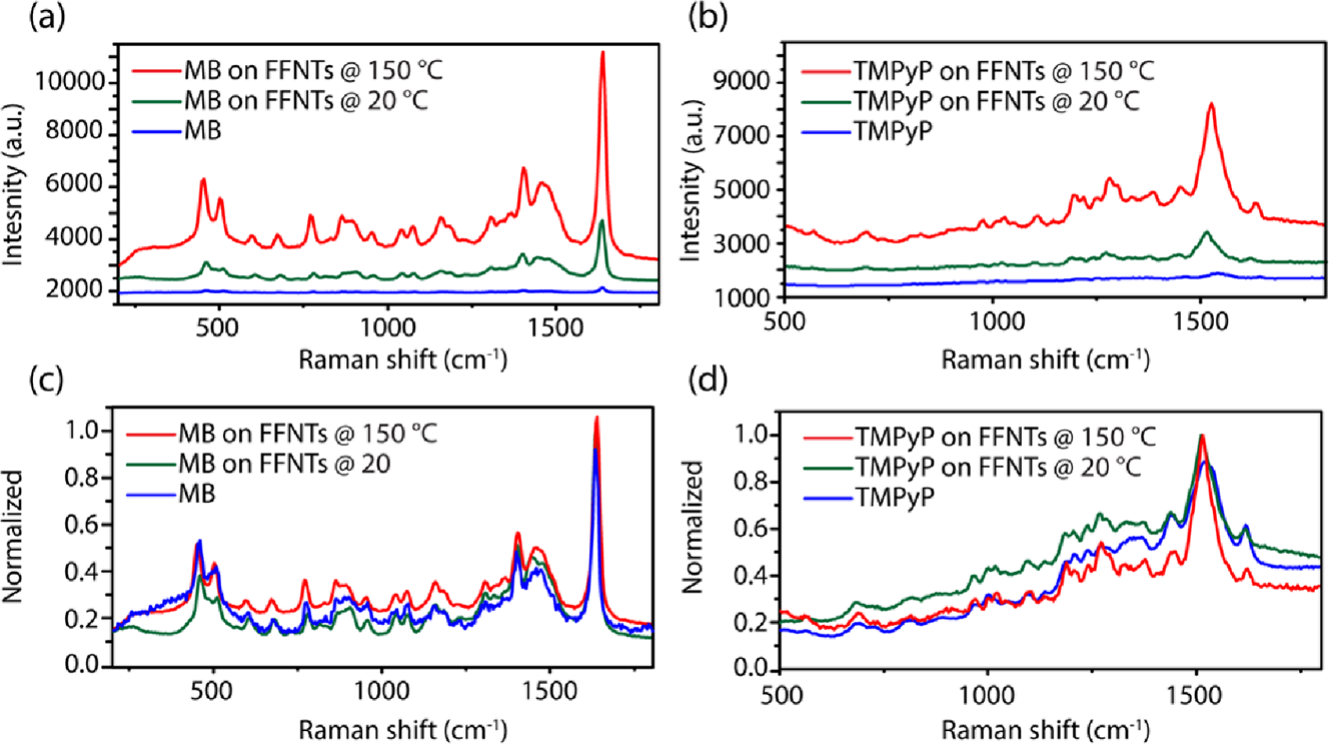
(a) PSERS of MB at a concentration of 10^–5^ M on pristine and annealed FFNTs. (b) PSERS of TMPyP at 10^–5^ M on pristine and annealed FFNTs. (c,d) Normalized spectra of the data presented in (a,b).

Peptide semiconductor-enhanced Raman scattering (PSERS) spectra from methylene blue (MB) and meso-tetra (*N*-methyl-4-pyridyl) porphine tetrachloride (TMPyP) at a concentration of 10^–5^ M for FFNTs annealed at different temperatures are shown in Figures 2 and S8–S10. PSERS intensities from both probe molecules increased as the temperature increased from 20 to 200 °C. However, at 200 °C, the peak-to-peak ratio decreased in comparison with that at 150 °C due to a high background signal (Figure S9). Such broad background signals have been reported for annealed zinc oxide.^8^

The PSERS peaks for MB on FFNTs annealed at 150 °C (Figures 2 and S9) were clearly visible and detectable. Characteristic bands including ν(C–C) ring stretching at 1560 cm^–1^, ν(C–N) symmetric and asymmetric stretching at 1433 cm^–1^, and the δ(C–N–C) skeletal deformation mode at 448–500 cm^–1^ all match with the literature.^33,34^ Similarly, increased PSERS from TMPyP on FFNTs annealed at 150 °C was observed (Figure 2b–d). Bands located at 1249 cm^–1^, C–C stretching at 1535 and 1451 cm^–1^, as well as the 1172 cm^–1^ C–H in-plane bending band are in good agreement with previous reports.^33,34^ The peak-to-peak intensity (PSERS vs Raman signal in the absence of FFNTs) for MB (at 1560 cm^–1^) and TMPyP (at 1535 cm^–1^) showed the greatest enhancement for FFNTs prepared at 150 °C, with enhancement factors of ∼7 and 6, respectively. Pristine FFNTs also led to PSERS in comparison with the probe molecule alone on a coverslip (Figure 2a). The peak-to-peak intensity of the 1535 cm^–1^ band increased ∼3-fold for MB on the FFNT in comparison with that of MB only on the coverslip, whereas TMPyP yielded a ∼2-fold increase of the 1553 cm^–1^ band.

**Figure 3:**
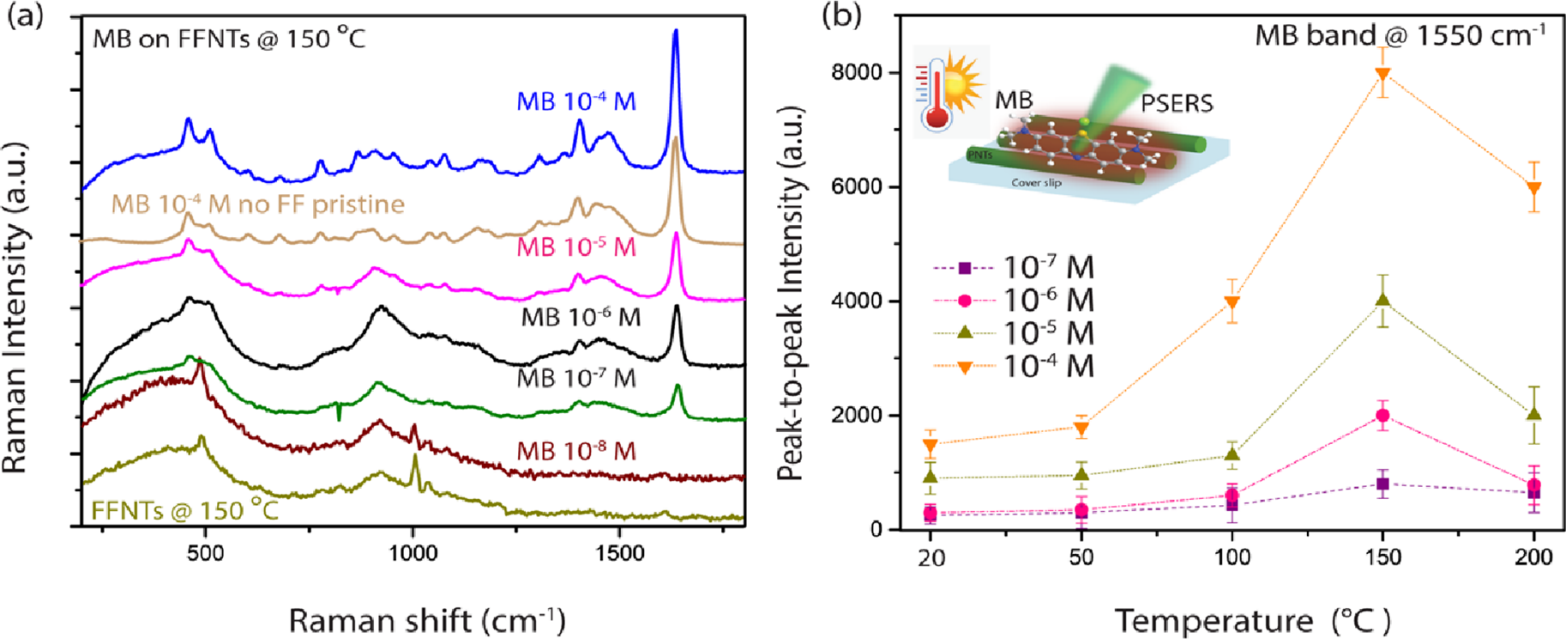
(a) PSERS of MB at concentrations of 10^–4^ to 10^–8^ M on FFNTs annealed at 150 °C; the detection limit is 10^–7^ M. Also shown are spectra of annealed (150 °C) FFNTs in the absence of MB. (b) Plot of relative scattering intensity from MB at ∼1550 cm^–1^ as a function of temperature for different MB concentrations.

MB could be detected down to a minimum concentration of 10^–7^ M using FFNTs annealed at 150 °C (Figure 3a,b). Evidence for such effects was found for a number of other probe molecules [such as methylene red (MR), crystal violet (CV), and methylene green (MG)] (Figures S11 and S12) in addition to MB. These molecules also show strong enhancement in the Raman signal strength when on annealed FFNTs (Figures S9 and S10).

The variation in PSERS from MB on FFNTs annealed at 150 °C was determined from multiple locations per sample to be 15, 17, and 19% of MB at concentrations of 10^–4^, 10^–6^, and 10^–7^ M, respectively (Figure S13), indicating that the FFNT template provides reproducible results.^35,36^

### Enhancement Mechanism

**Figure 4:**
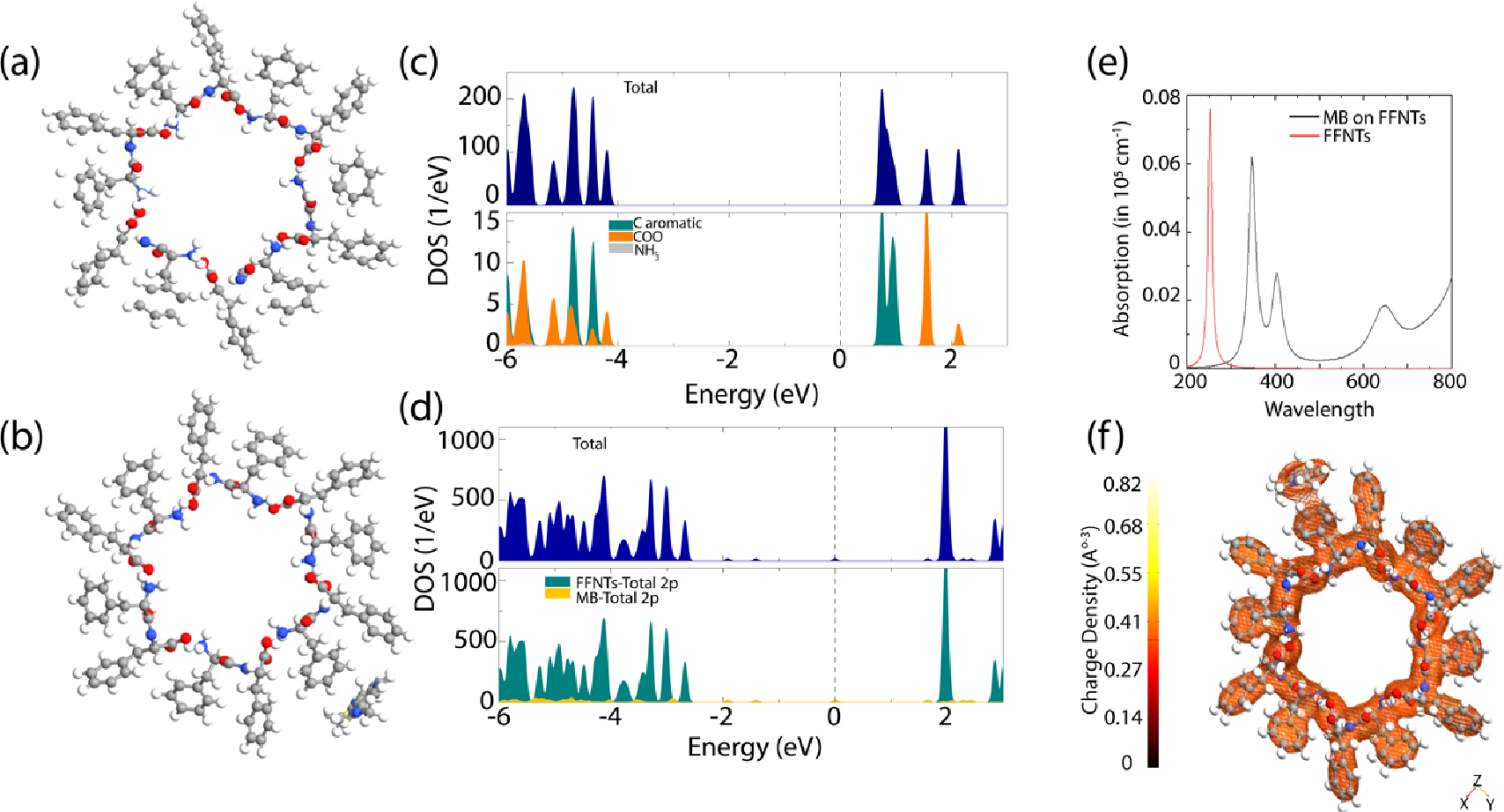
Schematic illustrations of the (a) FFNT unit and (b) MB adsorbed on FFNT [C (gray), H (white), N (blue), O (red), and S (yellow) are shown in a ball-and-stick model]. Calculated total and projected DOS of the (c) pristine FFNT and (d) MB on FFNT (only the 2p states are represented in the figure). (e) Simulated optical absorption spectra from the FFNT without and (b) with MB. (f) Electron charge density maps for MB on FFNT.

Theoretical calculations of an absorbed probe molecule on an FFNT demonstrate that changes in the electronic states occur. Simulations were performed for an FFNT hexagonal supercell (Figures 4a,b and S15; see Supporting Information) with and without absorbed MB molecules. According to the density of states (DOS) of the FFNT in (Figures 4c,d and S14), the HOMO is mainly composed of 2p orbitals of COO groups, whereas the lowest unoccupied molecular orbital (LUMO) of the CB is mainly composed of 2p orbitals from the FFNT’s aromatic groups. Moreover, the band structure of the FFNT exhibits a large direct band gap at gamma point [*k* = (0,0,0)] (Figure S14a), which agrees with the experimental results. For comparison with the projected DOS, the HOMO and LUMO are shown in Figure S15. Obviously, the HOMO is mainly around COO groups, whereas the LUMO is localized in an alternate fashion, consistent with the analysis of the projected DOS. For MB, the HOMO and LUMO are illustrated in Figure S15, which reveals that the HOMOs are localized on the area of the aromatic framework. However, the LUMOs are also concentrated on the aromatic group, with little delocalization. The calculated HOMO–LUMO energy gap of MB is 3.51 eV. The FFNT adsorbed with MB has significantly restructured its electronic band structure. Analysis of Figures 4d and S14b indicates that the adsorption of MB onto the FFNT introduces some molecule-originated electronic states inside the FFNT band gap region (Figure S14). Compared to the FFNT, the valence and the CB of MB on the FFNT system shift in the direction of higher energies. Besides, the occupied molecule states emerge inside the band gap upon the adsorption of MB, leading to a decrease in the band gap of the pristine FFNT. The phenomenon proves that MB can regulate and control the band structure of the FFNT. The band gap reduction caused by the adsorbed MB makes the FFNT more conducive to electronic transfer. Moreover, when MB interacts with the FFNT, 2p orbitals of MB not only alter the band edges of both the valence band (VB) and CB but also provide the forming energy states within the band gap, leading to a smaller band gap (Figure 4d). The absorption coefficients of the pristine FFNT and the MB adsorbed on the FFNT system are calculated and plotted in Figure 4e. The absorption edge of the FFNT adsorbed with MB is red-shifted. This also results from the narrowing of the band gap, in agreement with experimental observations (Figures S5–S12). To visualize the charge transfer, we illustrated the charge density plot of MB adsorbed on the FFNT (Figure 4f). Analysis of this plot suggests that the charge density iso-surface of 0.36 e/Å^3^ shows a relatively large overlap between the densities of MB and FFNT, indicating the existence of an electrostatic interaction. The plot of Figure 4eis evidence that the charge transfer involved is between the N(CH_3_)_2_ groups of MB and the aromatic groups of the FFNT. To investigate the FFNT with and without absorbed MB at a temperature of 150 °C, we first performed a molecular dynamics simulation based on both heating and cooling runs through a simulated annealing protocol. The simulated structure of the FFNT at 100 ps (Figure S17a) shows a change in the FFNT. Moreover, the potential energy of the FFNT system oscillates around a constant value of −1347 eV with a significant fluctuation magnitude (Figure S17b), showing the thermal stability of the new obtained structure. The band structure of the FFNT structure at 150 °C is calculated and shown in Figures S18 and S19. This shows that the CB and VB edges are shifted to lower and higher energies, respectively, producing a decrease in the FFNT band gap to 4.05 eV from 4.9 eV for the pristine FFNT. The changes in the electronic states in the FFNT following thermal annealing potentially support additional enhancement in the Raman signal, relative to when pristine FFNTs are used. The electronic states formed in the FFNTs following annealing at 150 °C may provide additional pathways for charge transfer processes, enhancing the chemical factor by strengthening the polarizability tensor and further altering the electron density distribution of the molecule, leading to the enhancement of the Raman signal.^9,24^

**Figure 5:**
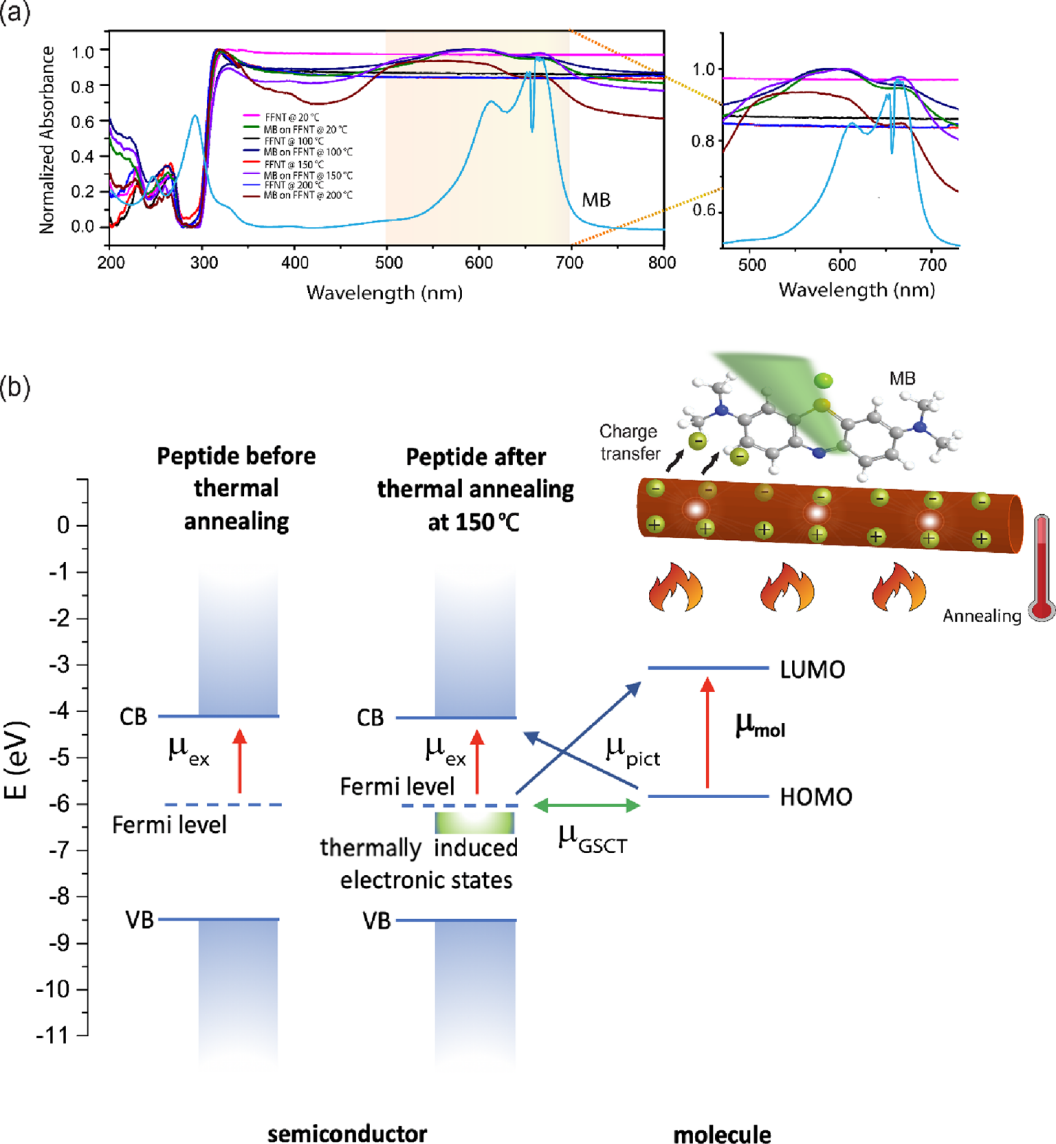
UV–vis measurements of annealed FFNTs on a glass coverslip with and without MB. (b) Proposed mechanism of PSERS and a band gap diagram to explain the possible charge transfer process between FFNTs and MB.

The increase in PSERS intensity after annealing can be attributed to several complementary effects. First, an apparently increased surface area [visible in SEM images (Figures 1bi–iii and S1)] provides more opportunity for probe molecule attachment^37−40^ and potentially higher Raman signals. Second, as shown in Figure 1biv, there was an associated change in wettability from hydrophilic to less hydrophilic with increasing temperature, as mentioned earlier. The increase of contact angle helps to artificially reduce the spreading of the probe molecule solution compared to the pristine case, yielding higher local concentrations and hence higher Raman signals.^8^ Third, charge transfer processes^41−46^ arising from the interaction between the probe molecule and the FFNT can also be enhanced. Optical absorption data (Figures 5, S20, and S21) for MB and TMPyP on the annealed FFNTs at 150 °C show that following the addition of probe molecules to FFNTs, there is a red shift of ∼10 nm for the band located at ca. 320 nm, near the valance band edge. Finally, we have observed from FTIR measurements that as the temperature increases, additional hydrogen bonds form (Figure 1).^39^ The significant enhancement in PSERS can be explained by the charge transfer effect induced by hydrogen bonds.^45^ These results suggest the possibility that hydrogen bonding transfers charge from the VB of FFNTs to the LUMO of MB, and due to the resulting potential difference, the charge will continue to transfer through the system. Therefore, the introduction of annealed FFNTs into the field of PSERS not only expands the PSERS substrate scope but also provides a new avenue for exploring the PSERS mechanism. In addition, the introduction of hydrogen bonds has become an important guide for the study of possible charge transfer and the structure of composite systems.^45^ It should be noted that the resonance effect also contributes to the overall enhancement in PSERS intensity from both MB and TMPyP on pristine and annealed FFNTs as both molecules are in resonance with the laser excitation wavelength when placed on the FFNTs, having absorption bands in the visible reason, as illustrated in Figures 5 and S22. It should also be noted that the chemical enhancement mechanism for other organic semiconductor platforms has been identified as the efficient π-orbital overlap of the semiconductor and analyte molecules’ LUMOs.^2,4^ Further work is needed to obtain direct experimental evidence for similar effective spatial overlap in the FFNT–analyte systems studied here.^41−43^

The band gap for annealed FFNTs (Figure S5) was estimated to be reduced from 4.4 eV (the value for FFNTs before annealing) to 3.86 eV. The Raman excitation laser (532 nm) at 2.33 eV has the energy to enable a ground-state charge transfer transition (μ_GSCT_) between the MB’s HOMO (5.67 eV) and the FFNT’s VB or work function (6.2 eV) or alternatively, or in parallel, a photo-induced charge transfer transition (μ_PICT_) between the MB’s HOMO and the annealed FFNT’s CB (3.9 eV) can occur. The strong chemical interaction between the two materials is outlined in our theoretical calculations (Figures 4 and S15–S19c) that support the existence of a charge transfer process between the semiconductor (FFNTs) and the analyte molecule under study, resulting in strong coupling between VB and LUMO or CB and HOMO, leading to different pathways for charge transfer processes to occur and hence improved chemical enhancement in SERS.^2−4^ Optical absorption data (Figures 5, S20, and S21) for MB on the annealed FFNTs at 150 °C show that following the addition of MB to FFNTs, there is a red shift of ∼10 nm for the band located at ca. 320 nm, near the VB edge. Additionally, the band at ca. 575 nm potentially arises from electronic states arising from analyte molecule–peptide (MB–FFNT) interactions. These optical absorption characteristics support the theoretical calculations (Figure 4) showing strong analyte molecule–peptide interactions.

**Figure 6:**
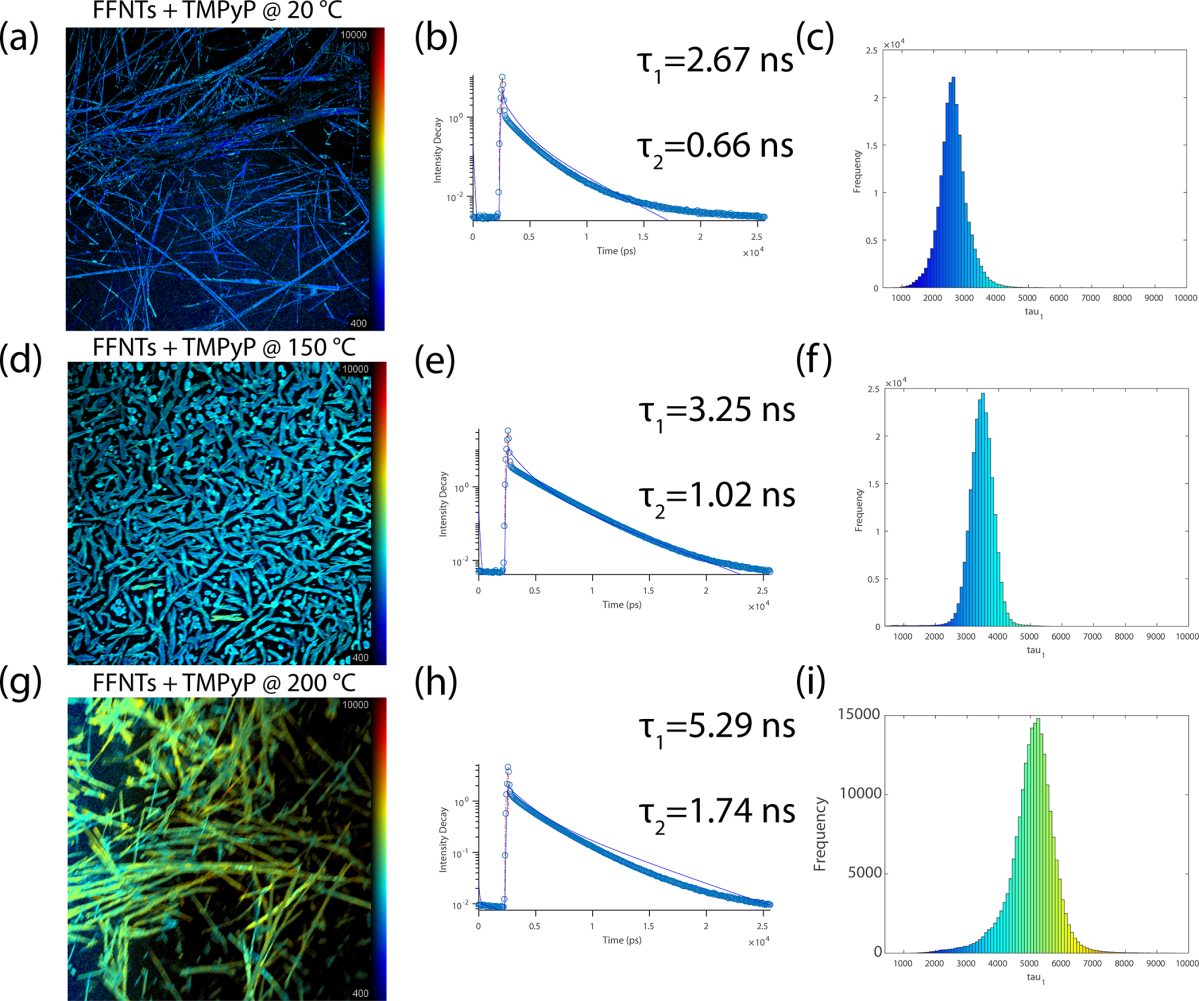
(a,d,g) Fluorescence microscopy images of TMyP 10^–4^ M on annealed FFNTs (20–200 °C). (b,e,h) Fluorescence decay curves along with the lifetimes calculated. (c,f,i) Lifetime histograms obtained for TMPyP on FFNTs annealed at 20, 150, and 200 °C, respectively. An excitation wavelength of 532 nm was used for all the measurements. A double exponential fit was applied to acquire values for the fluorescence lifetimes.

In order to better understand the mechanism responsible for the Raman enhancement of the probe molecules that are deposited on the annealed FFNTs (20–200 °C), fluorescence lifetime imaging (FLIM) by multidimensional time-correlated single-photon counting was performed (Figure 6). FLIM reveals that the fluorescence lifetimes for the probe molecule TMPyP above FFNTs increase from τ_1_ = 2.67 and τ_2_ = 0.66 ns at 20 °C to τ_1_ = 3.25 and τ_2_ = 1.02 ns at 150 °C and τ_1_ = 5.29 and τ_2_ = 1.74 ns at 200 °C. The increase in lifetime from molecules on the annealed FFNTs is due to a semiconductor-induced charge transfer process, allowing the extended lifetime for the excited singlet state between the FFNTs and probe molecule.^32^ Our result is in good agreement with literature reports,^32,35^ in which the presence of the FFNTs can potentially stabilize the excited state of the photosensitizer or dye molecules due to the charge transfer process. For longer fluorescence lifetimes, more energy is available to be transferred to molecules and hence enhance the PSERS intensity.^32^

Changes in fluorescence lifetime caused by the thermal annealing of the FFNTs could be associated with the presence of residual water incorporated into the hydrophilic channel of the FFNTs as well as increasing hydrogen bonding with heat.^18−21^ Studies have shown that the presence, interaction, and distribution of water molecules in the central hydrophilic channel of FFNTs lead to splitting of the CB and VB and hence alignment of the water molecules’ dipole moments and larger dipole moment of the overall structure.^15,29^ Thus, the presence of water molecules and additional hydrogen bonding in the nanochannel of the FFNTs would increase the stability of TMPyP in the excited state. This leads to an increase in the probe molecules’ dipole moment and potentially results in a stronger PSERS signal.

FFNTs annealed at 150 °C show a spectral red shift in the fluorescence emission peak compared to that of the pristine FFNTs (Figure 1). This red shift is attributed to a thermally induced rearrangement of the FFNTs’ bonds and formation of additional hydrogen bonds as shown from the FTIR data, Figure 1. Such changes in FFNT bonds lead to the formation of new energy states.^30−32^ These newly formed states in FFNTs at 150 °C potentially support charge transfer between the FFNT and the probe molecule, resulting in strong PSERS signal enhancement (Figures 2 and 3). The lifetime histogram at 150 °C shows a narrow distribution compared to that of FFNTs at 200 °C (Figure 6f). Studies have shown that the narrow distribution of the average lifetimes reflects a more homogeneous environment, meaning that the molecules seem to be rather homogeneously distributed above the annealed FFNTs.^22^

Additional heating of FFNTs at 200 °C results in a change in the fluorescence lifetime of TMPyP (τ_1_ = 5.29, τ_2_ = 1.74 ns at 200 °C) compared to that at 150 °C. This increase in lifetime at 200 °C is accompanied by a blue shift in the fluorescence emission wavelength (Figure 1) that could be attributed to changes in the electronic states present in FFNTs following heating at 200 °C. This potentially results in a reduction in charge transfer between the probe molecules and the FFNTs at 200 °C relative to that at 150 °C, reducing the PSERS signal enhancement (Figure 2).

**Figure 7:**
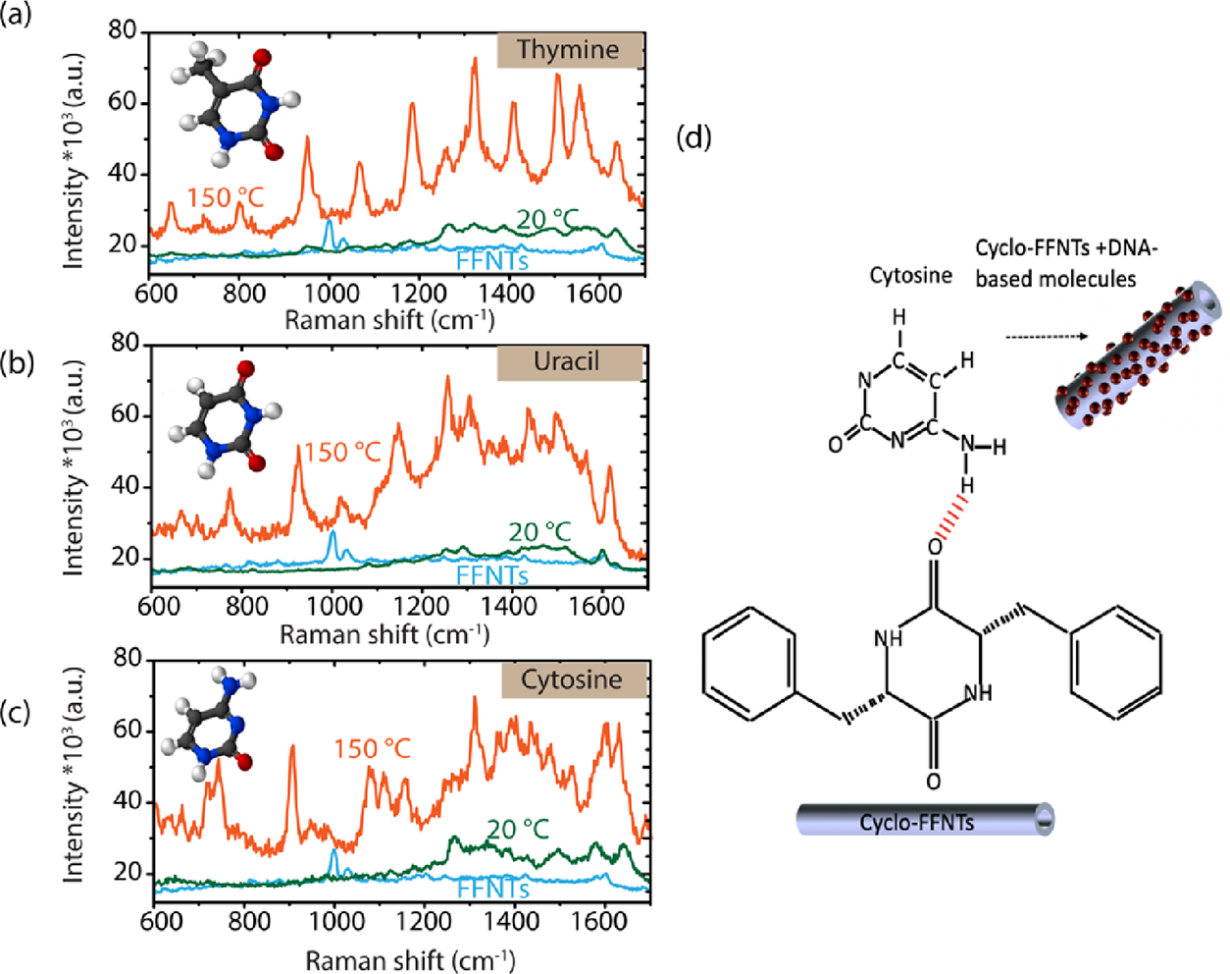
PSERS spectra from (a) thymine (10^–5^ M), (b) uracil (10^–5^ M), and (c) cytosine (10^–5^ M) on pristine FFNTs and FFNTs annealed at 150 °C. (d) Schematic showing the hydrogen bonding interaction between DNA-based molecules and the FFNTs.

To further widen the use of FFNT templates with the annealing process in biomolecules and biomedical applications, the nucleobase molecules thymine, uracil, and cytosine were investigated at a concentration of 10^–5^ M, as illustrated in Figure 7. It should be noted that DNA-based molecules are nonresonant with the laser excitation wavelength (532 nm), having a band gap in the UV region, as illustrated in Figure S22. Therefore, these probe molecules are of interest to study for comparison with MB and TMPyP and focus only on the semiconductor–molecule charge transfer process. Undertaking measurements on DNA-based molecules enabled us to study both resonance and nonresonance Raman scattering. We have successfully detected all reported thymine, uracil, and cytosine bands, and the peak positions are in line with previous reports.^16,47,48^ Peaks from thymine such as bands at 1470 cm^–1^ (CH_3_ bending), 1397 cm^–1^ (N_1_–H bending and N_3_–H), 1221 cm^–1^ (C_5_–C_9_), and 785 cm^–1^ (ring breathing mode) dominate the PSERS spectra (Figure 7a). Strong bands were recorded for uracil at 1489 cm^–1^ (stretching C_6_–N_1_, C_4_–C_5_, and C_2_=O) and 1630 cm^–1^ (stretching C_2_=O, C_4_=O, N_1_–H bending, and C_5_–H) (Figure 6b). Additionally, cytosine bands at 1591 cm^–1^ (NH_2_ bending), 1544 cm^–1^ (N_3_–C_4_–C_5_), 1422 cm^–1^ (N_1_–H, C_5_–H, and C_6_–H), 1228 cm^–1^ (C–N), and 1038 cm^–1^ (ring breathing mode) were detected (Figure 6c). Under optimized conditions (at 150 °C for 40 min), a 6–7-fold increase in the PSERS signal was observed (compared with that of pristine FFNTs). FFNTs bind with nucleobase molecules through hydrogen bonding, as shown in Figure 7, increasing the chemical interaction between the substrate and the nucleobases and potentially increasing the resulting Raman signal.^16,49^ Detection of nucleobases demonstrates the capability of the annealed FFNT template to extend the detection limit of FFNTs and could be potentially applied in many Raman-based biomedical diagnostic schemes. The strong enhancement of the PSERS signal of the mononucleotides may result from the strong adsorption of the molecule on the FFNT substrate. This strong absorption can result in perturbations of the electronic and geometrical structure of the probe molecule that changes the Raman cross-sections of the vibrational modes of the molecules with respect to those of the free molecules, resulting in stronger Raman intensity.

## Conclusions

Metal-free PSERS substrates with highly sensitive detection capabilities have been successfully fabricated employing annealed FFNTs as an organic semiconductor template. We demonstrate that bioinspired semiconducting FFNTs heated through a reported structural transition can support Raman detection of 10^–7^ M concentrations for a range of molecules. The enhancement is attributed to the introduction of electronic states below the CB following annealing of the FFNT, which facilitates charge transfer to the analyte molecule; however, further work is required to establish the exact chemical enhancement mechanism. The significant enhancement in FLIM observed from the annealed FFNTs could be attributed to the enhancement of the excited-state lifetime of the probe molecules. Fluorescence lifetime contrast-based imaging provides new insights into the field of semiconductor-induced enhancement via charge transfer processes. These findings highlight that organic FFNT semiconductor-based materials can serve as platforms for enhanced Raman scattering for chemical sensing and provide a new avenue for the design of highly efficient semiconductor PSERS substrates for chemical detection applications.

## Materials and Methods

### Template Formation and Oven Heating

FFNTs were formed by dissolving l-diphenylalanine peptide (Bachem) in 1,1,1,3,3,3-hexafluoro-2-propanol (Sigma-Aldrich) to a concentration of 100 mg/mL, which was further diluted in deionized water to a final concentration of 2 mg/mL. The oven was preheated to 100, 150, or 200 °C, and then the samples (FFNTs on coverslips) were placed in the oven for ∼40 min.

### Probe Molecule Solutions

To prepare TMPyP solutions, TMPyP powder (T40125, Frontier Scientific) was diluted with deionized water to a range of concentrations from 10^–4^ to 10^–7^ M. MB, thymine, cytosine, and uracil were all sourced from Sigma-Aldrich, Ireland, and similarly diluted with deionized water to a range of concentrations from 10^–4^ to 10^–8^ M.

CV 1% aqueous solution (CAS Number: 548-62-9) was diluted in distilled water down to the concentration of 10^–5^ M. MG (zinc chloride salt, ∼85%; CAS:7114-03-6) was dissolved in distilled water at initial concentration 10^–2^ M and then diluted down to 10^–5^ M using deionized water. MR (ACS reagent, crystalline; CAS Number 493-52-7) was prepared in chloroform (analytical reagent grade; CAS: 67-66-3) at an initial concentration of 10^–2^ M and then diluted down to 10^–5^ M using chloroform. The probe molecule solutions were drop-cast (40 μL) on the pristine and annealed FFNTs and left to dry for 2–3 h prior to Raman measurements.

## Supporting Information Available

Additional data and experimental details including substrate characterization using SEM and EDX mapping, XPS analysis of annealed FFNTs, UV–vis and band gap determination as a function of temperature and with probe molecules, PL as a function of laser power, CD analysis, Raman spectra of FFNTs as a function of temperature, and DFT calculations (PDF)
