# Supplementary material for: Structural Transition-Induced Raman Enhancement in Bioinspired Diphenylalanine Peptide Nanotubes
Source: ACS Appl Mater Interfaces. 2022 Mar 7;14(10):12504–14. doi: 10.1021/acsami.1c22770 (PMC8931724; doi:10.1021/acsami.1c22770)
Supplement: Supplementary file 1 — am1c22770_si_001.pdf [file am1c22770_si_001.pdf]

Supporting Information

Structural transition-induced Raman enhancement in bioinspired diphenylalanine peptide nanotubes

Sawsan Almohammed [a, b], Agata Fularz [a], Mohammed Benali Kanoun [c], Souraya Goumri-Said [d], Abdullah Aljaafari [c], Brian J. Rodriguez * [a,b], and James H. Rice * [a]

[a]*School of Physics, University College Dublin, Belfield, Dublin, D04 V1W8, Ireland*
[b]*Conway Institute of Biomolecular and Biomedical Research, University College Dublin, Belfield, Dublin, D04 V1W8, Ireland*
[c]*Department of Physics, College of Science, King Faisal University, P.O. Box 400, Al-Ahsa 31982, Saudi Arabia*
[d]*Physics Department, College of Science and General Studies, Alfaisal University, P.O. Box 50927, Riyadh, 11533, Saudi Arabia*

1. Substrate characterization using SEM and EDX mapping

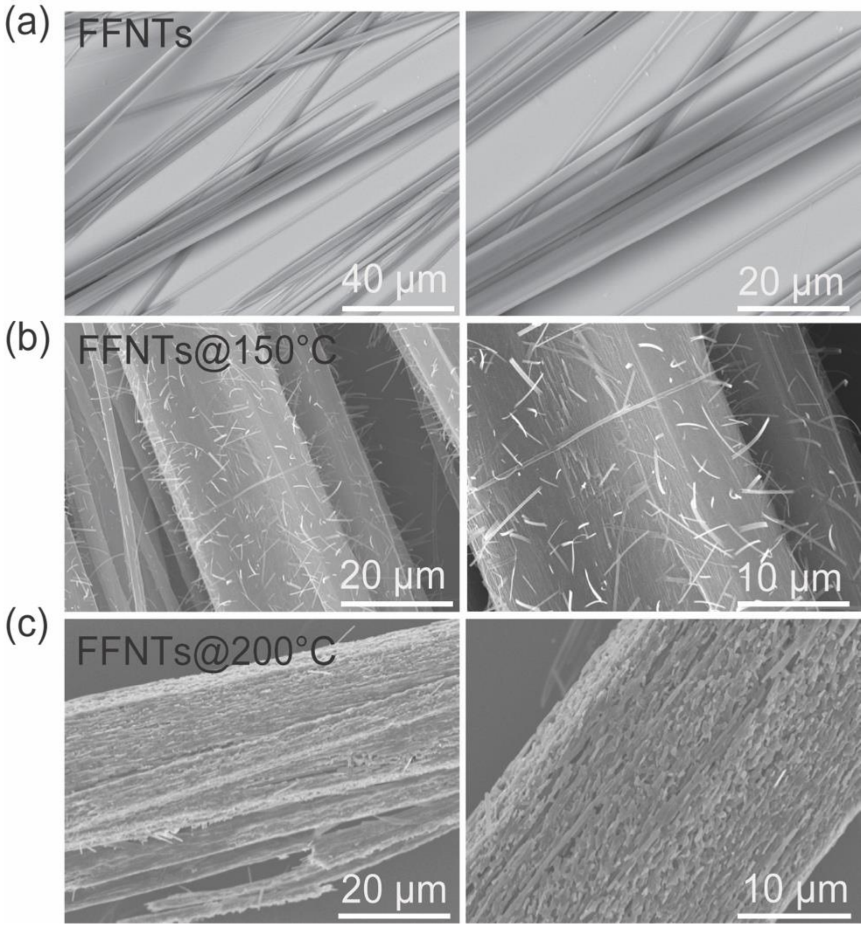

Figure S1. SEM images of FFNTs (a) before and after annealing at (b) 150 °C and (c) 200 °C.

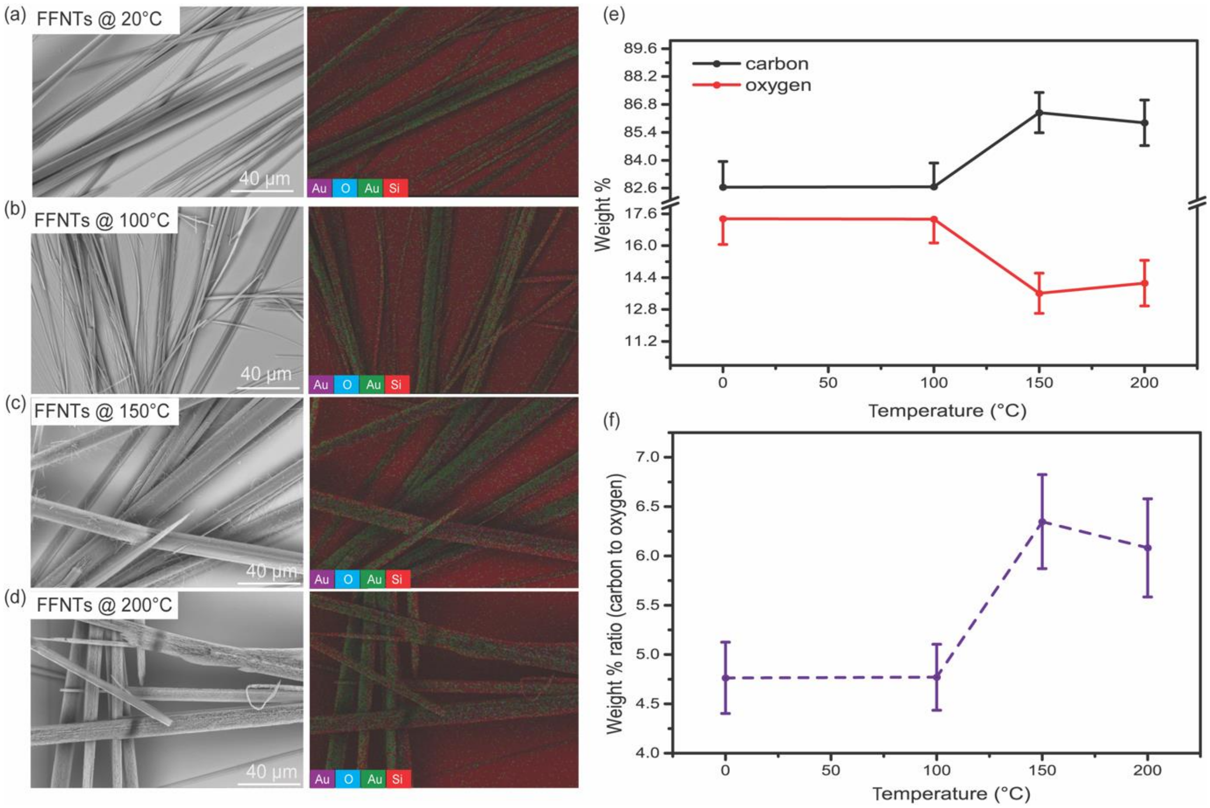

Figure S2. (a–d) SEM images and EDX mapping of FFNTs annealed at different temperatures. (e,f) Ratio of carbon and oxygen atoms as a function of temperature.

2. XPS analysis of annealed FFNTs

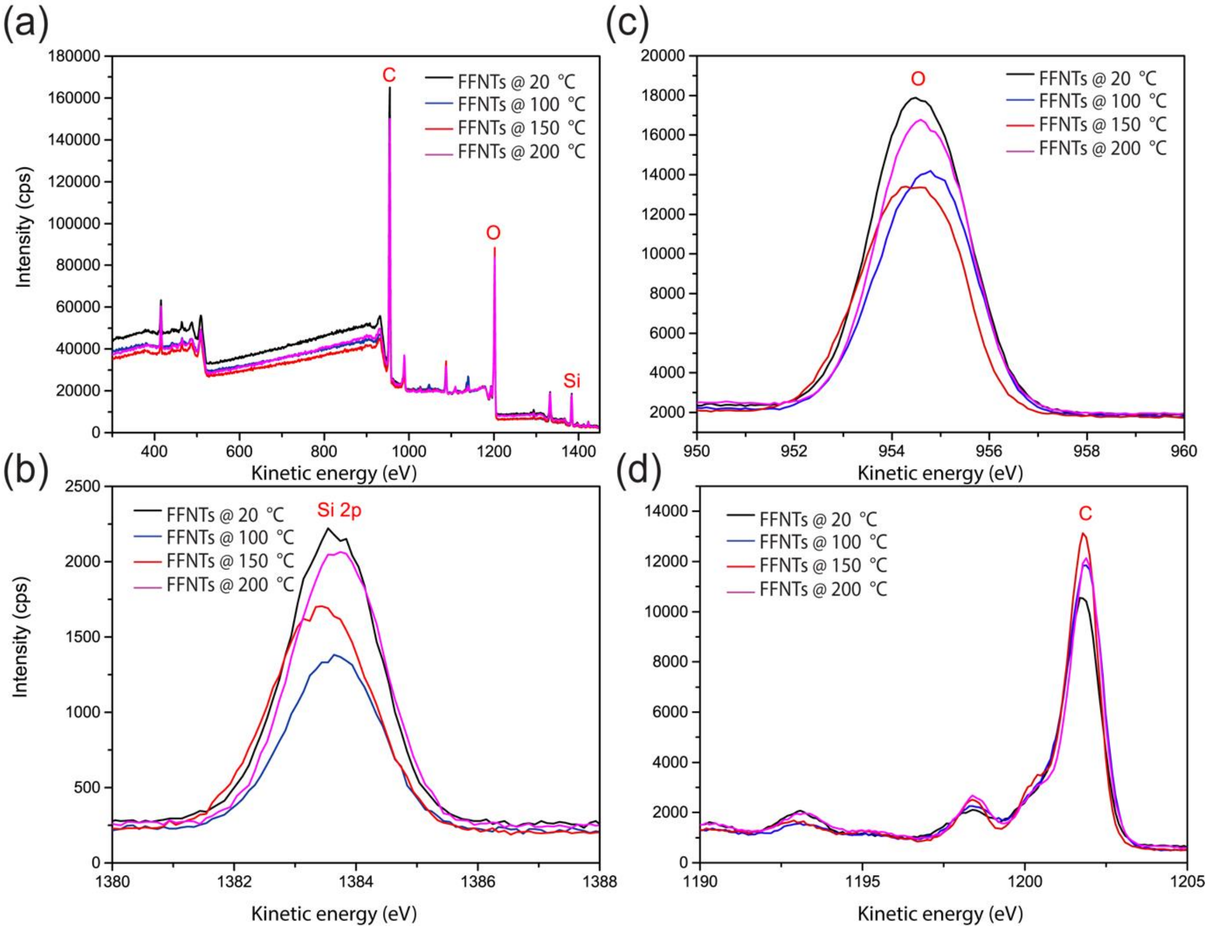

Figure S3. (a–d) XPS spectra for annealed FFNTs at different temperatures.

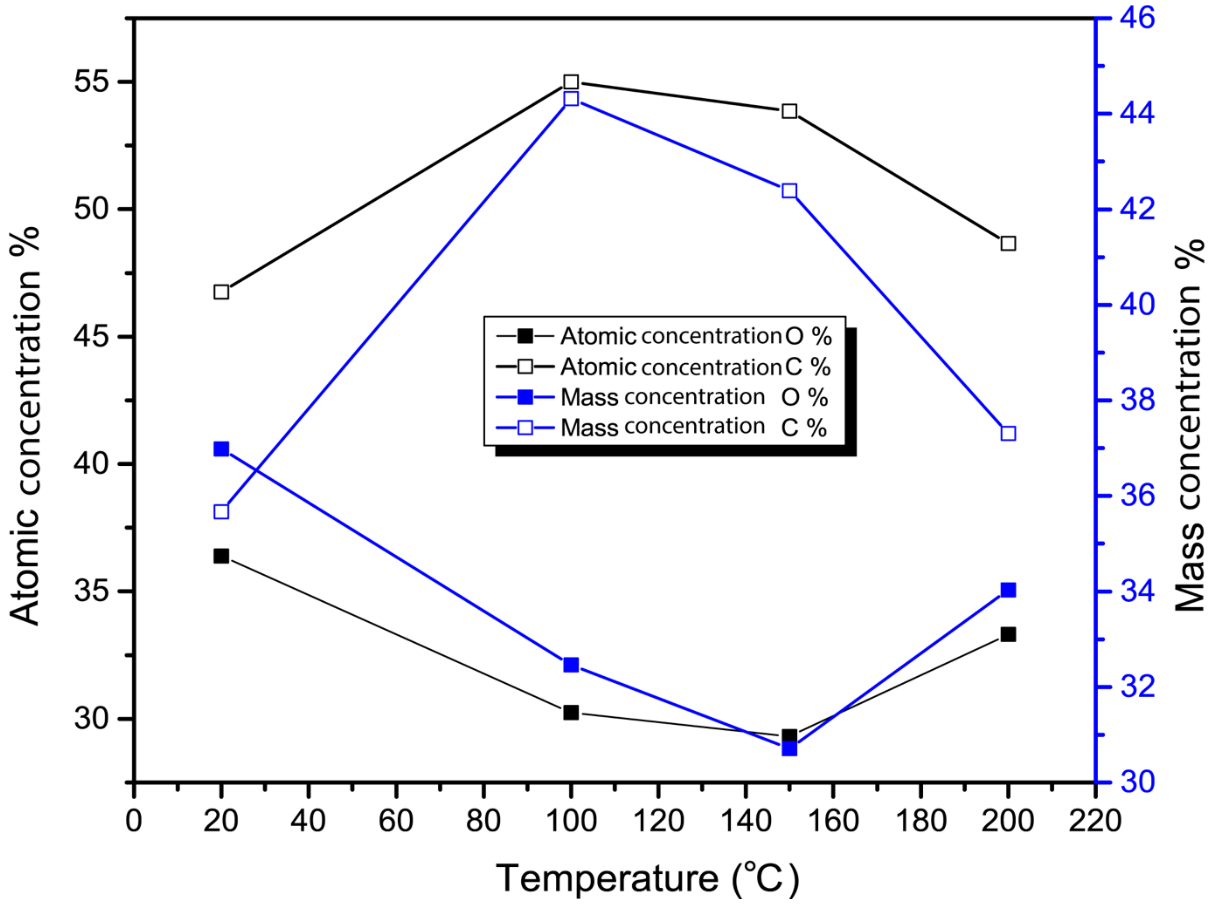

Figure S4. Atomic concentration and mass concentration of O, N, and C of FFNTs annealed at different temperatures from 20–200 °C (determined from XPS spectra).

3. UV-vis and band gap determination as a function of temperature

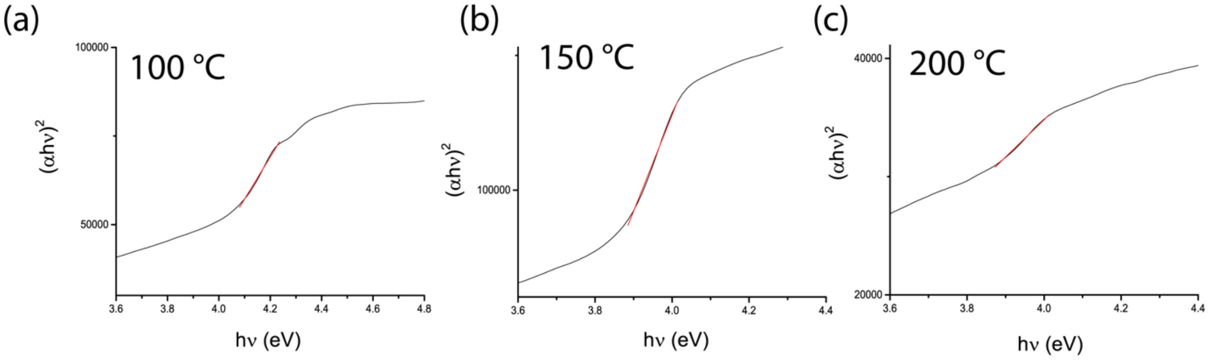

Figure S5. Band gap determination from UV-vis measurements of FFNTs annealed at (a) 100, (b) 150, and (c) 200 °C.

4. Photoluminescence of FFNTs as a function of temperature

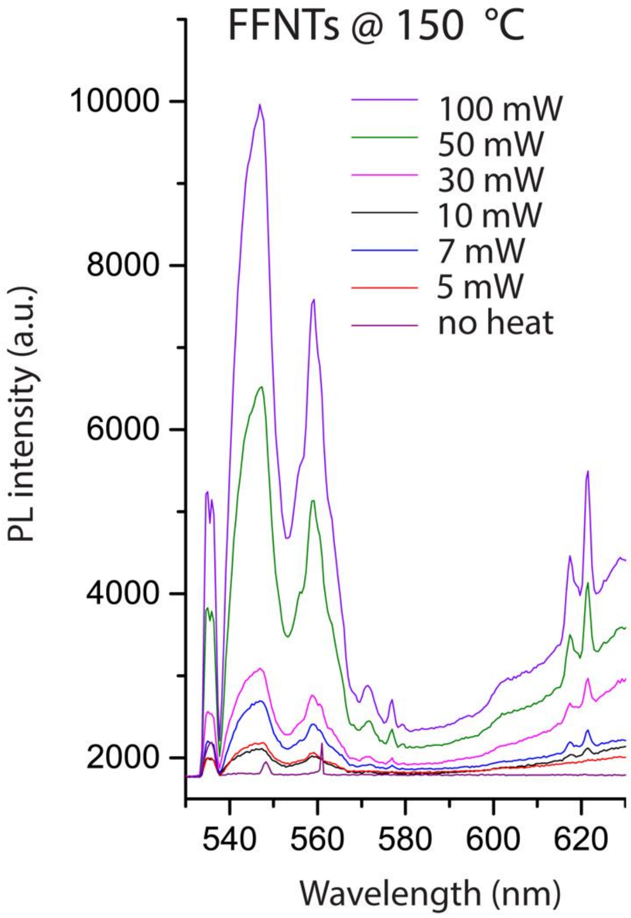

Figure S6. Photoluminescence (PL) spectra (excitation wavelength 532 nm) of pristine and annealed (at 150 °C) FFNTs at different laser powers up to 100 mW, showing the stability of the FFNTs at high laser illumination.

5. Circular dichroism (CD) analysis

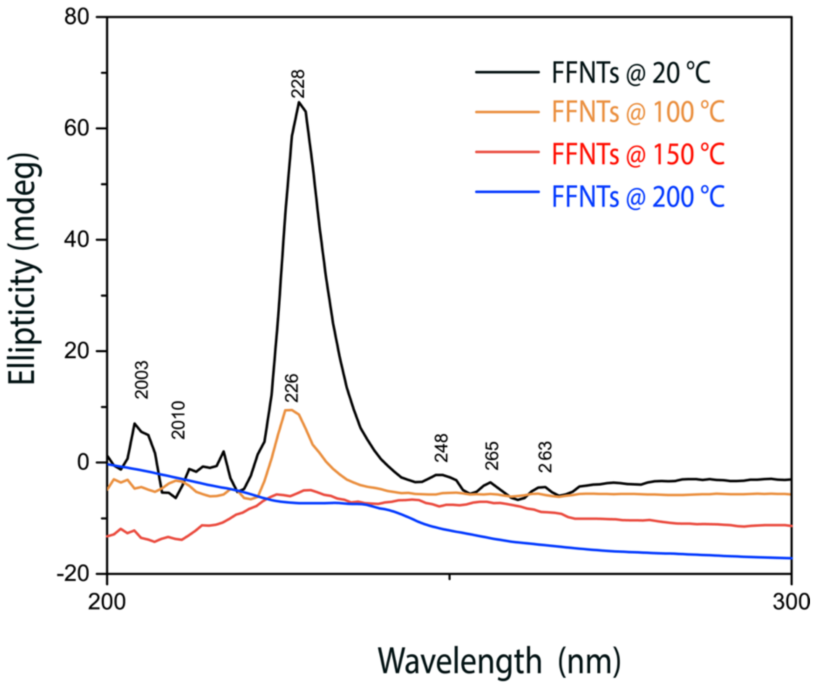

Figure S7. CD spectra vs temperature of the studied FFNTs nanostructures showing the variation of the peptide secondary structure in the region of reconstructive phase transition. The FFNTs CD spectra show that as the temperature increases, the sign of the ellipticity of FFNTs nanostructures changes and becomes negative, suggesting a β-turn to β-sheet transition.

The positive CD spectra of FFNTs (Fig. S7) in the native phase are known to be influenced by aromatic side chains.[1] The results of previous studies, which demonstrated a positive CD spectrum of phenylalanine-based nanostructures, were ascribed to a β-turn conformation. After heating to elevated temperatures, the CD spectrum of the formed FFNTs-fibers becomes negative. This is completely consistent with the behavior of FFNTs nanostructures when they undergo a thermally induced phase transition at 150−200 °C.[1] The bands correspond to n−π* and π−π* transitions with temperatures, the sign and ellipticity of the CD bands remains approximately constant and negative. Thus, we conclude that heating at temperatures between 150-200 °C leads to an inversion of the sign of CD ellipticity for all of the inspected peptide ensembles.

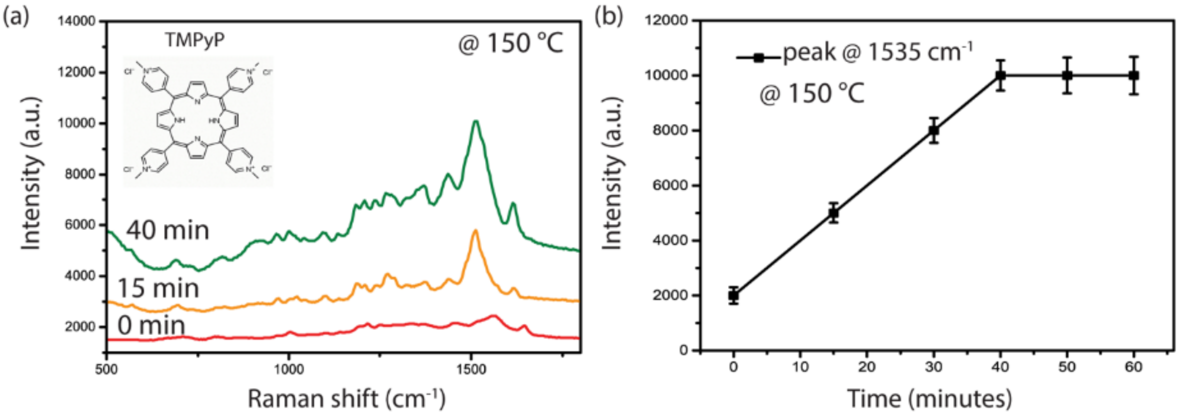

Figure S8. (a) Raman measurements of 10^{-5} M TMPyP on FFNTs annealed at 150 °C for different durations. (b) Plot of relative intensity of the TMPyP band at 1535 cm^{-1} as a function of time at 150 °C.

As an initial study, PSERS was recorded from FFNTs annealed at 150 °C with different periods of time, varying from 15 to 60 minutes. The highest enhancement was observed following annealing for 40 minutes. Heating longer than this resulted in no further improvement in Raman signal strength from the probe molecule.

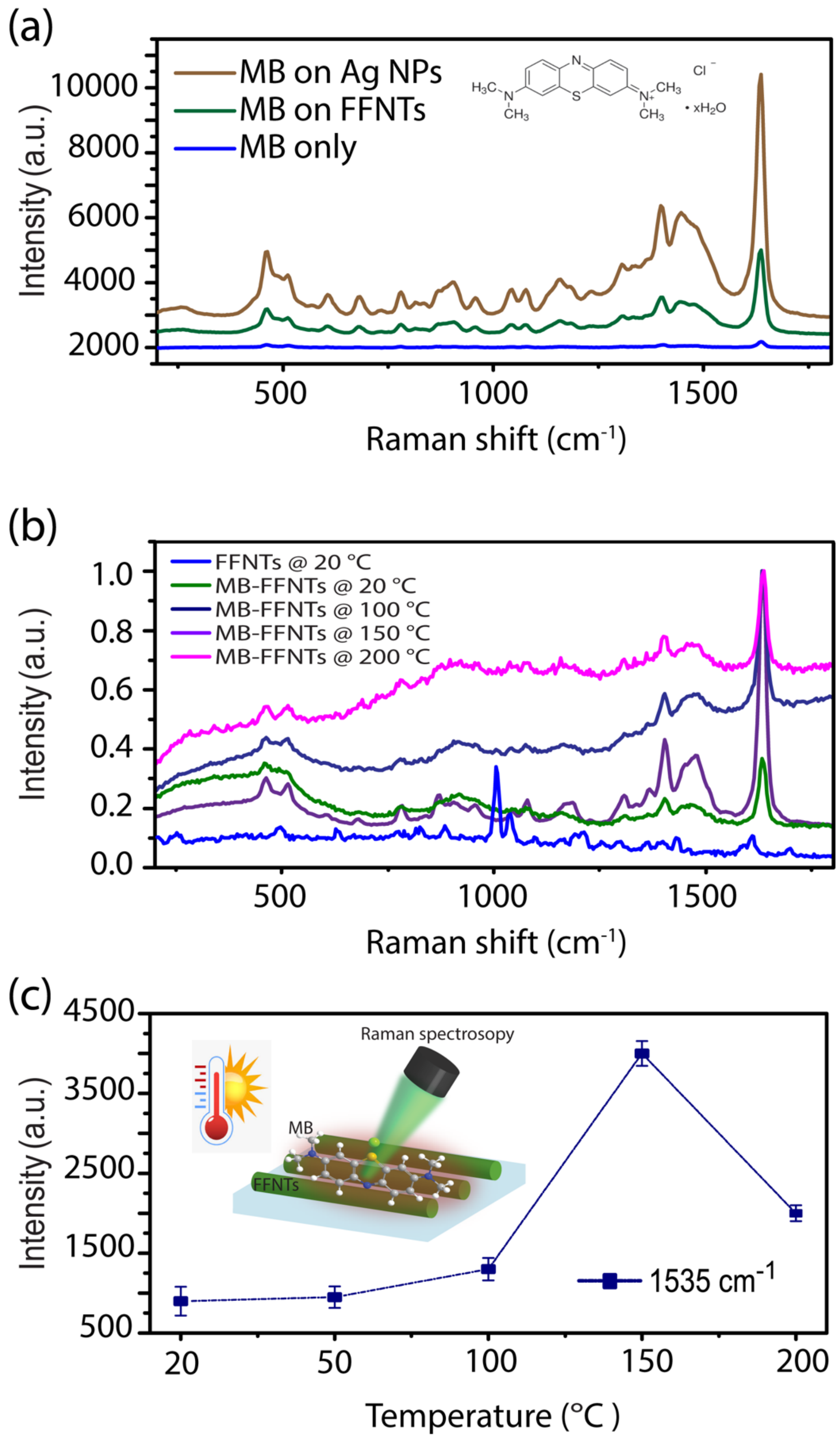
(a)
MB on Ag NPs
MB on FFNTs
MB only
Intensity (a.u.)
Raman shift (cm-1)
(b)
FFNTs @ 20 °C
MB-FFNTs @ 20 °C
MB-FFNTs @ 100 °C
MB-FFNTs @ 150 °C
MB-FFNTs @ 200 °C
Intensity (a.u.)
Raman shift (cm-1)
(c)
Raman spectrosopy
MB
FFNTs
1535 cm-1
Intensity (a.u.)
Temperature (°C)

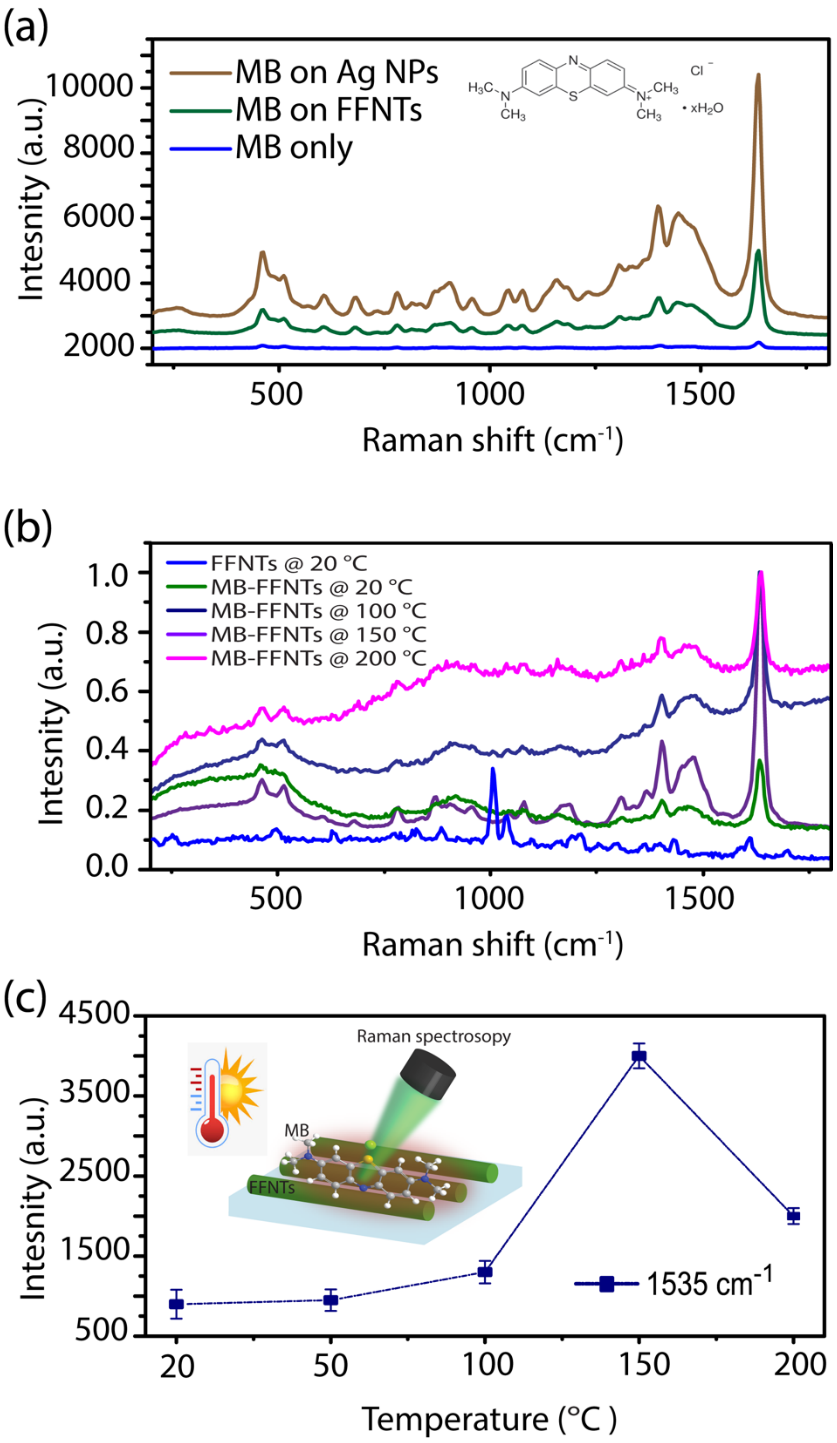

Figure S9. (a) Raman measurements of 10^{-5} M MB on pristine FFNTs in comparison with MB only and with Ag NPs, recorded at room temperature. (b) Normalized Raman of MB on FFNTs at different temperatures from 20–200 °C. (c) Plot of relative intensity (peak-to-peak) of the MB band at 1535 cm^{-1} as a function of temperature. The inset shows a schematic of Raman measurements of annealed FFNTs.

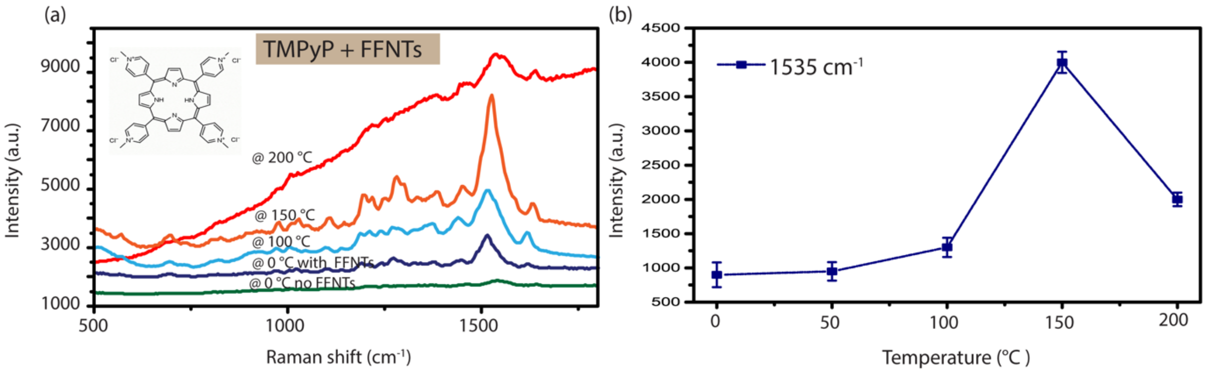

Figure S10. (a) Raman measurements of 10^{-5} M TMPyP on FFNTs annealed at different temperatures from 20–200 °C. (b) Plot of relative intensity (peak-to-peak) of the TMPyP band at 1535 cm^{-1} as a function of temperature.

Fig. S9 shows PSERS measurements of TMPyP at 150 °C. The presented data shows bands located at 1535 cm^{-1} (C−C stretching) and 1451 cm^{-1} (C−C stretching) for the probe molecule TMPyP as well as the 1172 cm^{-1} C−H in-plane bending mode, in good agreement with previous reports.[2]

6. PSERS measurements of methylene red (MR), methylene green (MG), and crystal violet (CV)

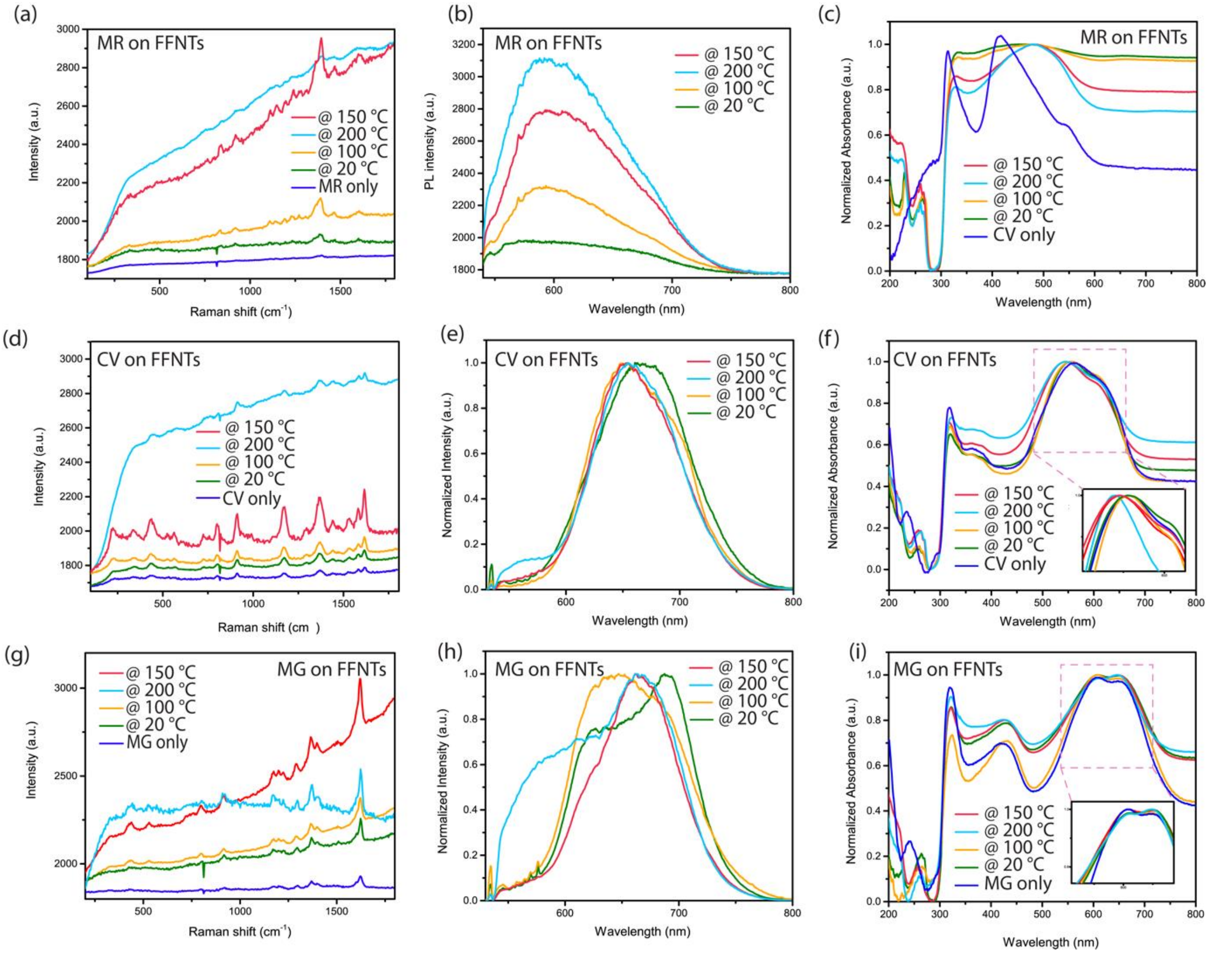

Figure S11. (a, d and g) PSERS of MR, CV, and MG at a concentration of 10^{-5} M on pristine and annealed FFNTs. (b, e, and h) Photoluminescence measurements of MR, CV, and MG at a concentration of 10^{-5} M on pristine and annealed FFNTs. (c, f, and i) UV-vis measurements of MR, CV, and MG on pristine and annealed FFNTs on a cover slip.

PSERS from methylene red (MR), crystal violet (CV), and methylene green (MG) at concentrations of 10^{-5} M on pristine and annealed FFNTs at different temperatures are shown in Fig. S11. PSERS intensities from all probe molecules increased as the temperature increased from 20−150 °C, ~7-fold, 7.5-fold, and 9-fold, respectively. However, at 200 °C, the peak-to-peak ratio decreased in comparison to 150 °C, due to a high background signal, similar observations were made for MB and TMPyP. The main characteristic peaks of CV at around 1616, 1368, 1174, 915, and 450 cm^{-1} are associated with in-plane stretching of the ring–C–C, N–phenyl stretching, in-plane bending of the ring–C–H, ring skeletal vibration of radical orientation, and out-of-plane deformation vibrations of the phenyl–C^{+}–phenyl.[3] SERS spectra of MR and MG were similar to those of CV because of their similar molecular structures, and only changes in the relative intensity among peaks were observed, such as for the band at 1395 cm^{-1} for MR, 1621 cm^{-1} for MG, and 1616 cm^{-1} for CV (Fig. S12). The characteristic Raman peaks of MG were observed in the high-frequency region. Namely, the strong Raman

band belonging to N–C bending and C–C stretching vibrations were seen at 1621 cm^{-1}. The bands at 1171 cm^{-1} and 1294 cm^{-1} can be assigned to the aromatic C–H in-plane bending vibrations and 1366 cm^{-1} can be assigned to the N–C stretching vibration coupled with the C–C and C–H in-plane motions at 1394 cm^{-1}. The band shown at 914 cm^{-1} can be attributed to the ring skeletal radial vibration.[4]

The Raman peak at 1395 cm^{-1} in the spectra of MR is the deformation vibration of the aromatic C–N bonds. The Raman peak at 1141 cm^{-1} in the spectra of MR is the deformation vibration of the aromatic C–C bonds. The Raman peak at 441 cm^{-1} in the spectra of MG is the deformation vibration of the conjugated aromatic C–C bonds. The peak at 484 cm^{-1} in the spectra of methylene blue is the deformation vibration of the C– N–C bonds.[5]

Fig. S11b, e and h show emission spectra of MR, CV, and MG on pristine and annealed FFNTs at different temperatures. It can be seen from all probe molecules used quenching of the fluorescence spectra at 150 °C, an indication of possible charge transfer in the system. Also, there was strong blue and red shifting of the PL bands from all molecules, which is subject for further future study. Similar shifting was observed in the absorption spectra of MR, CV, and MG on pristine and annealed FFNTs at different temperatures, recorded on cover slip Fig. S11b, e and h. MR shows an absorption band located at 432 nm (Fig. S12c), that was red shifted to ~530 nm on both on pristine and annealed FFNTs at different temperatures making it more in-resonance with the laser wavelength. However, both CV and MG blue shifted, making the systems more in-resonance with the laser excitation wavelength.

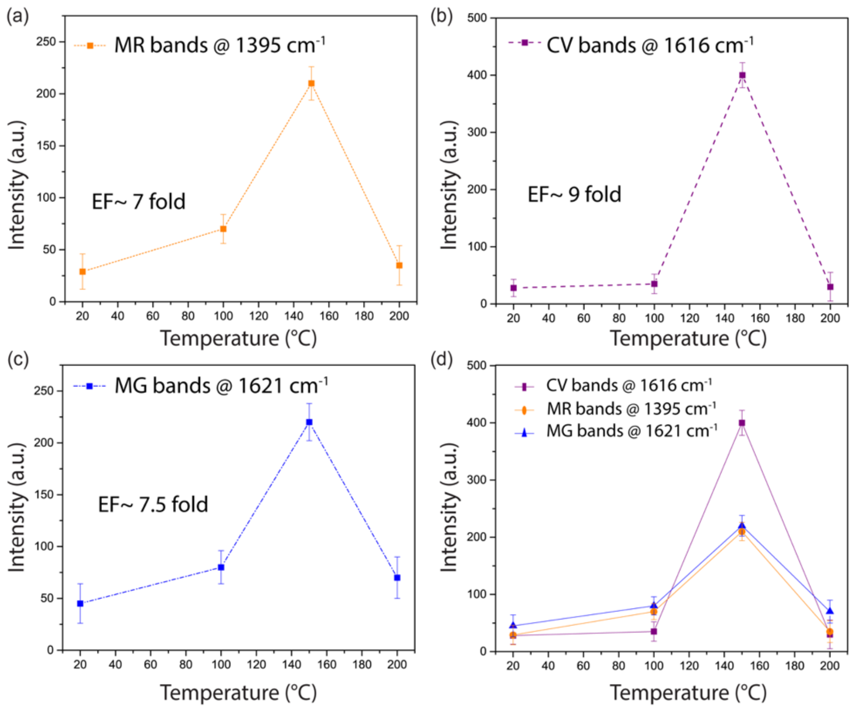

Figure S12. (a-d) Plot of relative scattering intensity from MR at ~1395 cm^{-1}, CV at 1616 cm^{-1}, and MG at 1621 cm^{-1} as a function of temperature on annealed FFNTs.

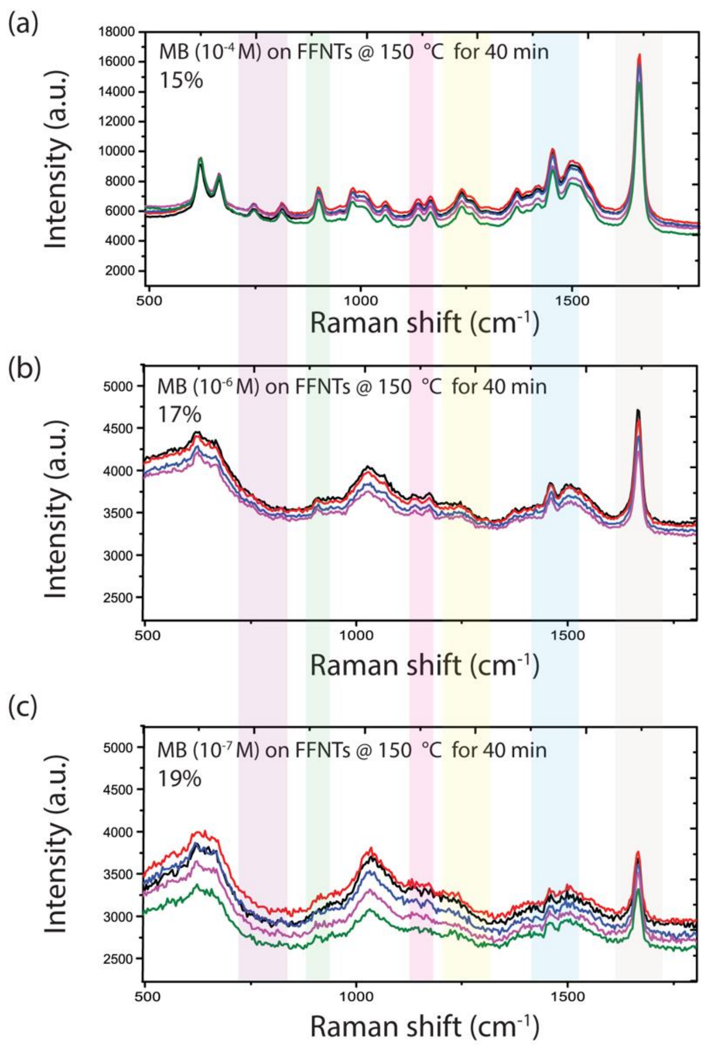

Figure S13. Uniformity test of MB at different concentrations: (a) 10^{-4} M, (b) 10^{-6}, and (c) 10^{-7} collected from ~5 locations on each sample of FFNTs annealed at 150 °C. There is a 15–19% variation of the Raman intensity of the band at 1550 cm^{-1}.

7. Structure modeling and DFT calculations

DFT was performed to explore the electronic properties and optical absorption spectra of FFNT, MB, and MB adsorbed on FFNT. As a starting point, a FFNT unit is cut from a FFNT initially based on structure obtained from X-ray data,[6,7] which is described by a hexagonal crystal structure (space group $P6_1$), as shown in Fig. S15. To be more specific, the FFNT was constructed from a 2 × 2 × 1 hexagonal supercell by removing the atoms external circle of 2.8 nm diameter, see Fig. 4a. The selected atoms and molecules from the hollow structure containing 258 atoms. Thus, ensuring that the FFNT contained a vacuum space of ~15 Å perpendicular to the FFNT axis (the z direction), which is sufficiently large to avoid the interactions between the FFNT walls under periodic boundary conditions. Our optimized equilibrium lattice constants for the FFNT in the lateral direction and in along the c axis are 24.2 Å and 5.48 Å, respectively, which well reproduced the measured X-ray diffraction values.[6,8] In the case of MB on FFTN complex, the approach adopted when modelling the complex system that includes the 1 × 1 × 3 supercell FFNT configuration adsorbed with MB.

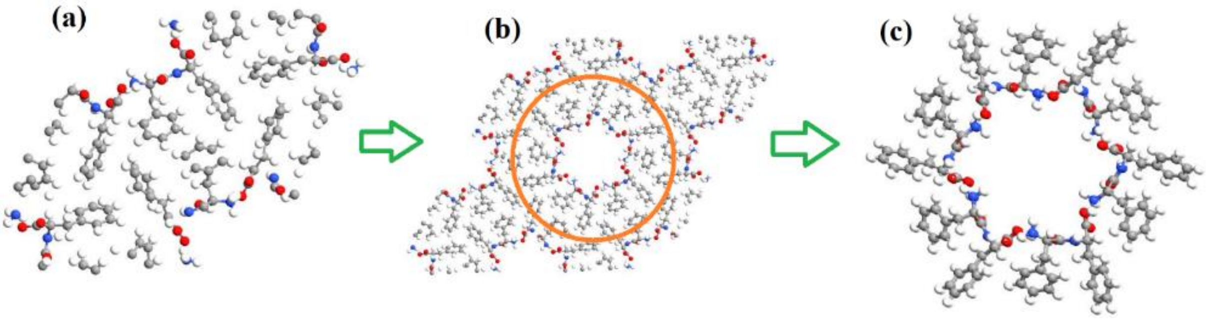

Figure S14. (a) FF crystal structure with space group $P6_1$. (b) Selected atoms and molecules for the formation of FFNT are shown with an orange circle. (c) The building block of FFNT.

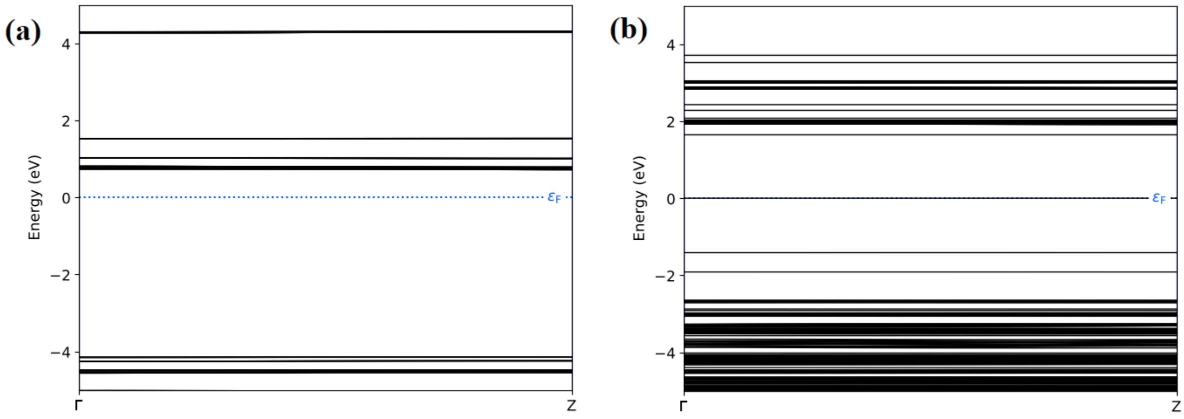

Figure S15. The electronic band structures of FFNT (a) without and (b) with MB.

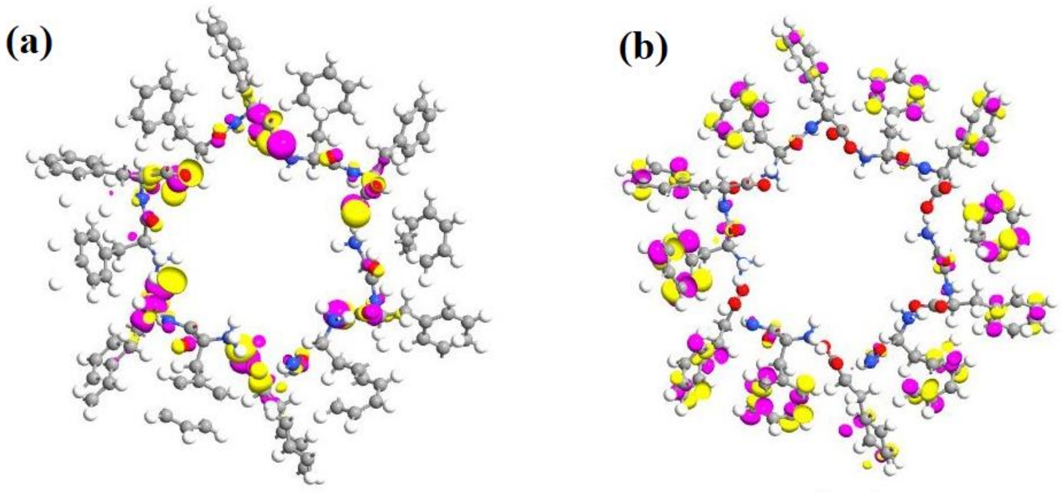

Figure S16. (a) HOMO and (b) LUMO charge densities of the FFNT. Isosurface value of 0.06 e/Å^3.

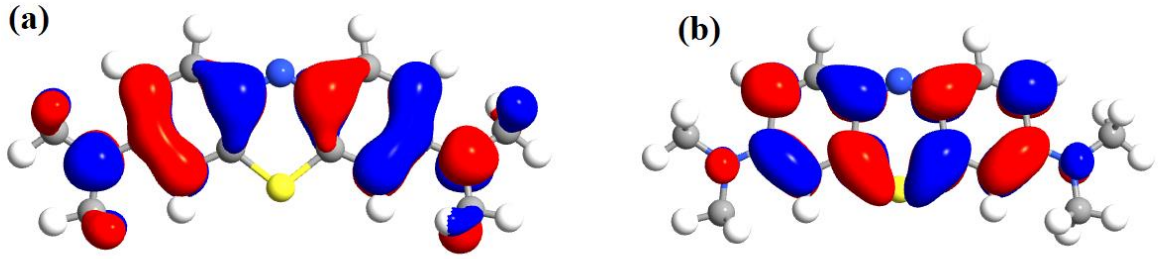

Figure S17. Illustration of the (a) HOMO and (b) LUMO charge densities of MB molecule. Iso-surface value of 0.12 e/Å^3.

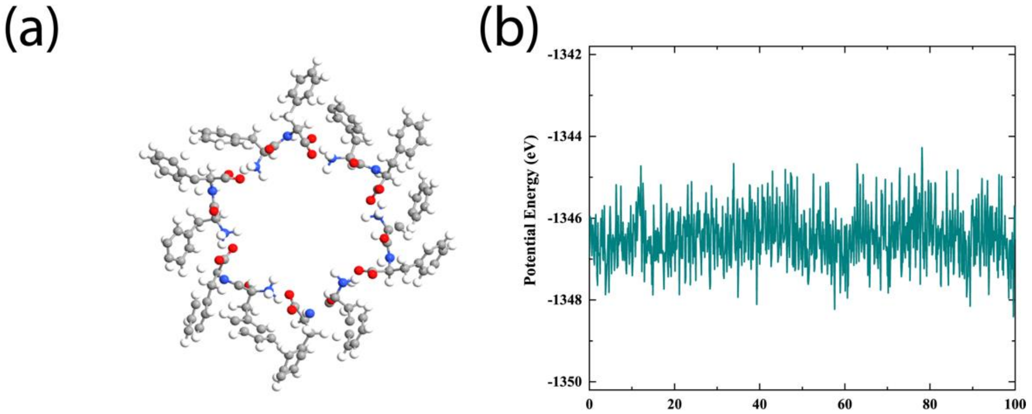

Figure S18. (a) Top view (b) potential energy fluctuations during MD simulation of FFNT after 100 ps at 150 °C.

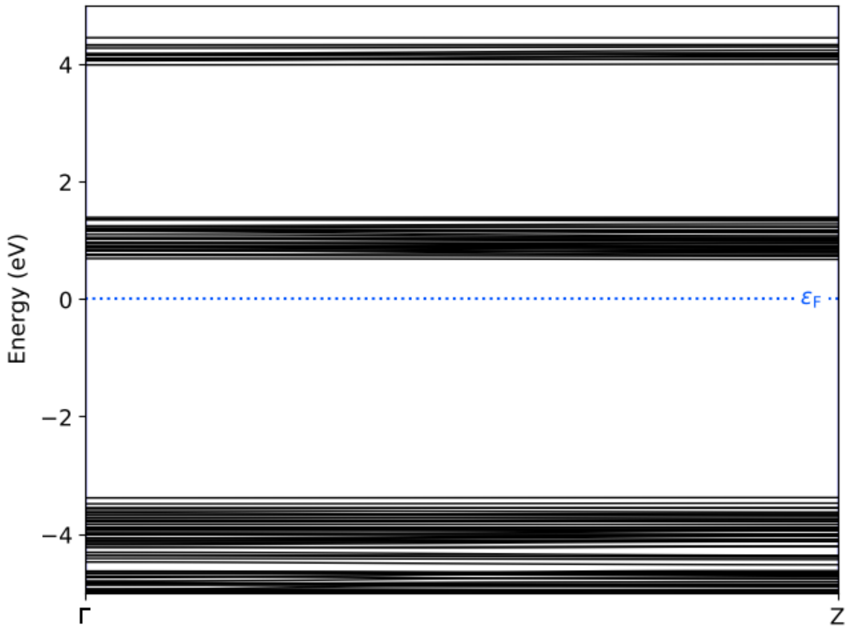

Figure S19. The electronic band structure of FFNT at 150 °C.

8. UV-vis measurements after probe molecule deposition

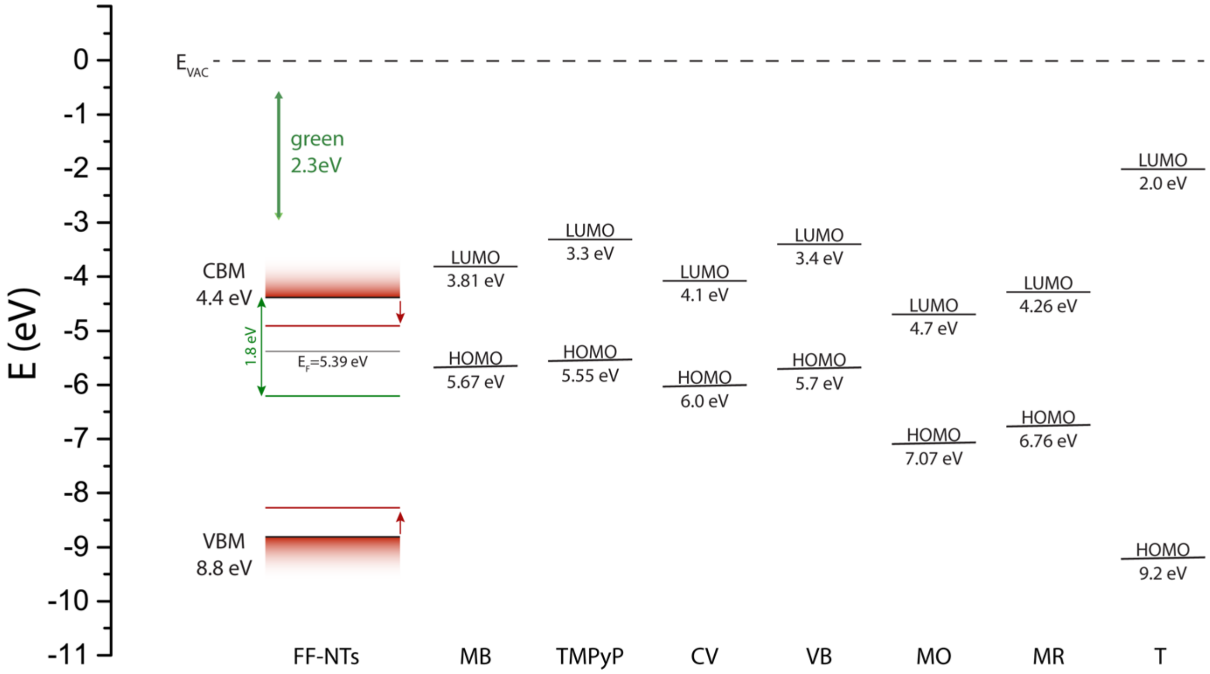

Figure S20. Band diagram of FFNTs to explain the possible charge transfer process between FFNTs and probe molecules used in this study, before and after annealing. The diagram shows thermally-induced band gap narrowing from 4.4 eV to 3.86 eV (red line and arrow) as observed from UV-vis measurements (Fig. S5). In addition, thermally-induced defect states are present 1.8 eV below the conduction band (from fluorescence measurements (Fig. 1)). These defect states are shown as a green line.

Following the process reported in Fig. S5 to estimate the band gap from UV-vis data after adding the probe molecule MB, we have found that there is significant reduction of the band gap (~3.3 eV) of annealed FFNTs, even lower than having FFNTs only (~3.86) without the analyte. Such a reduction in the band gap of FFNTs after depositing the analyte molecules highlight the strong chemical interaction between the two materials in agreement with our theoretical calculations Figs. 4 and S16-S21. Such interactions lead to improved charge transfer between the semiconducting material (FFNTs) and the analyte molecule under study, resulting in strong coupling between VB and LUMO or CB and HOMO, leading to different pathways for charge transfer to occur and hence improved chemical enhancement in SERS.[9–11]

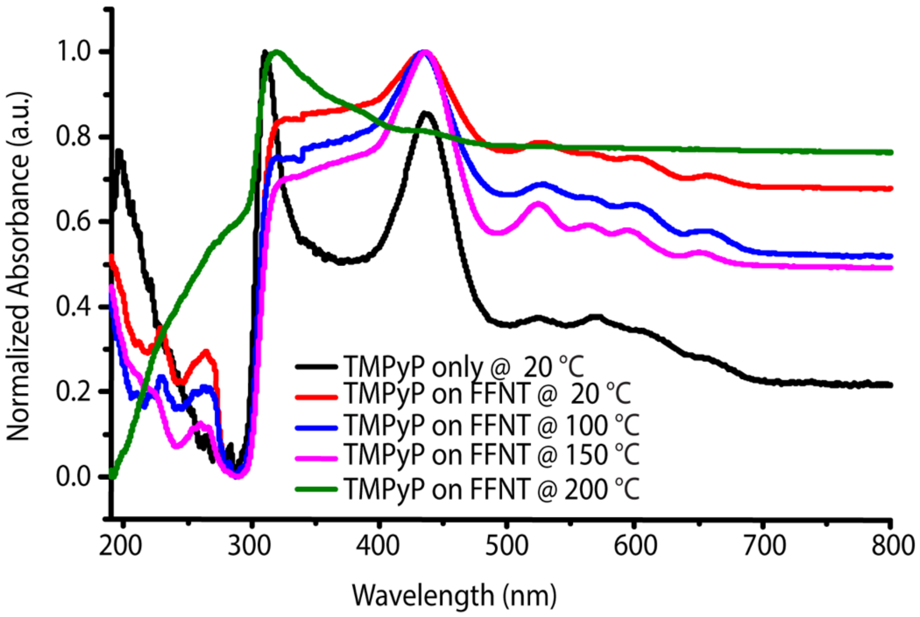

Figure S21. UV-vis measurements of annealed FFNTs on a cover slip with and without probe molecules.

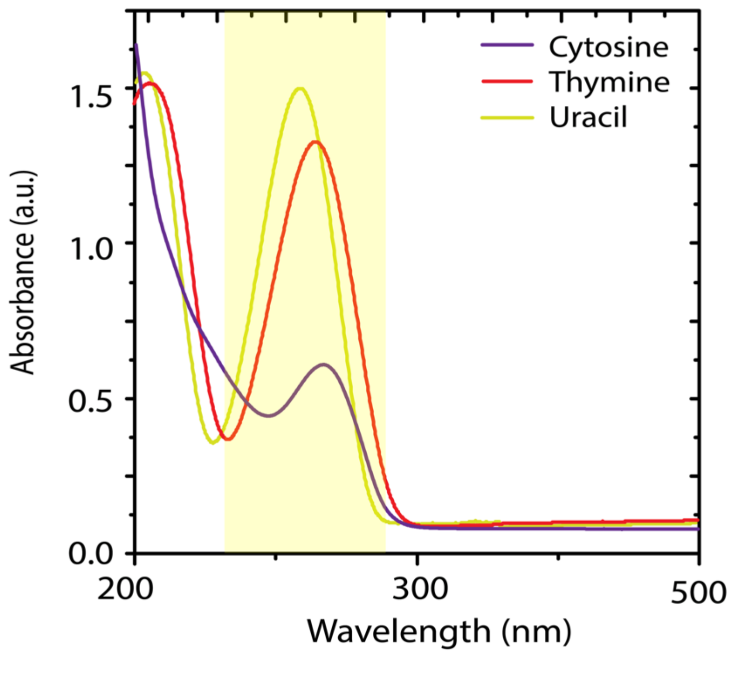

Figure S22. UV-vis measurements of cytosine, thymine, and uracil. These molecules have an absorption band in the UV region between 240–255 nm, which make them non-resonant with the 532 nm laser excitation wavelength.

Methods

Optical absorbance measurements (UV-vis)

The UV-vis spectra were recorded (V-650, JASCO, Inc.) over a 190–900 nm wavelength range. UV-vis was undertaken using a 1 nm step size, with a 1 nm bandwidth, and a 400 nm/min scan speed. Sample preparation for UV-vis was as described above. Briefly, the FFNTs on glass coverslips annealed at different

temperatures were prepared for UV-vis measurements to be undertaken, with and without the probe molecules.

Circular Dichroism (CD) Spectroscopy

Circular dichroism (CD) spectra were acquired on a spectropolarimeter (J-810, Jasco Inc.). Scans were recorded from 170 nm to 350 nm, at 5 nm bandwidth and 1 s integration time.

Fourier transform infrared (FTIR) spectroscopy

FTIR spectra were recorded using transmission and absorption modes (Alpha.Platinum-ATR, Bruker) using the same sample preparation as for UV-vis.

Contact angle measurements

A contact angle measuring system (DSA10, Krüss) equipped with a camera was used to measure contact angles of 10 µl droplets of deionized water placed on each sample.

Fluorescence imaging

Fluorescence confocal and fluorescence lifetime microscopy images were obtained using the Leica TCS SP8 confocal system using a white light laser set to 532 nm and an internal HyD GaAsP SMD detector. FLIM fit was performed using FLIM fit program 5.1.1 (global fitting of large fluorescence lifetime). A 10 x objective was used, and the samples were imaged through a coverslip in air, without the introduction of a mounting medium.

Scanning electron microscopy (SEM) and energy-dispersive X-ray spectroscopy (EDX) mapping

Scanning electron microscopy (JSM-7600F, JEOL, operated at 5 kV) was employed to characterize the pristine and annealed FFNTs. A thin (~8 nm) layer of gold was sputtered on the samples before SEM imaging (Hummer IV, Anatech USA). EDX mapping was performed using the same system at 10 kV and 10 nA.

X-ray photoelectron spectroscopy

The samples were analyzed by X-ray photoelectron spectroscopy (XPS) (Axis Ultra^{DLD}, Kratos, Ltd., UK) using an Al $K_α$ (1486.7 eV) X-ray source. All samples were outgassed for 12 h under ultra-high vacuum before analysis.

Raman and surface enhanced Raman spectroscopy

In order to record PSERS spectra, a bespoke Raman system was employed. This system consists of an inverted optical microscope (IX71) with a SP-2300i spectrograph (Princeton Instruments), and an EMCCD camera (IXON). The Raman excitation wavelength was 532 nm, which was fixed at ~5 mW incident laser power. The same system was used for photoluminescence measurements. The CCD camera was calibrated over the spectral window using toluene. Each spectrum shown is an average of spectra recorded from ten different spots on a sample unless otherwise stated. PSERS enhancement factor values were calculated by comparing the intensity of the appropriate MB peak (at 1560 cm$^{−1}$) and TMPyP (at 1535 cm$^{−1}$) MR peak at 1395 cm, CV 1616 cm^{-1}, Mg 1621 cm^{-1} measured in the PSERS experiments to the corresponding peak measured from normal Raman spectra of the probe molecules without the use of FFNT materials.

The SERS enhancement factor (EF) is given by:

EF= N_{vol} I $_{PSERS}$ /N_{surf} I_{Raman}

where N_{vol} and N_{surf} are the number of molecules probed in the sample and on the SERS substrates, respectively. I_{PSERS} and I_{Raman} are the corresponding PSERS intensities and normal Raman. Assuming the number of molecules are the same on the substrate as we are using the same drop size of materials. Both I_{PSERS} and I_{Raman} is peak to peak intensity therefore the baseline was subtracted from the initial peak intensity. The EF was calculated several times each time resulting in similar EF for each PSERS substrate.

Theoretical calculations

Our first principles calculation was conducted using Density Function Theory (DFT) as implemented in the QuantumATK software[11] using local combination of the atomic orbitals (LCAO) approach. The exchange correlation functional was conducted by Perdew, Burke, Ernzerhof (PBE) connecting with the generalized gradient approximation (GGA).[11] The norm-conserving PseudoDojo[12,13] pseudopotential with medium basis set and a mesh cut-off energy of 10^5 Ha was employed for describing the interaction between electrons and ions, and the valence electrons. The Brillouin region was performed by means of Monkhorst-Pack's special k-point grid of 1 × 1 × 4 for structural relaxation and 1 × 1 × 7 for electronic property calculations of FFNT and MB on FFNT. The calculation of self-consistent field (SCF) considered a tolerance limit of 10^{-6} Ha for energy convergence. The geometry structure and ion relaxations were carried out by using the limited-memory Broyden–-Fletcher–Goldfarb–Shanno (LBFGS) algorithm, including the force on each atom less than 0.05 eV/Å. The HOMOs and the LUMOs were evaluated by DFT through QuantumATK using the DFT-1/2 method.[14] They are constructed to determine the energy levels of the molecular orbitals. The employment of DFT-1/2 method was required to more precisely characterize the computed band gap.[15]

In addition, molecular dynamics (MD) simulations were performed using the reactive force field (ReaxFF) to examine the heating effect on FFNT systems through a simulated annealing process. The initial structure was heated to 200 °C during 50 ps and then slowly cool it down to 150 °C in 100 ps. The NPT Berendsen method was employed with a damping constant of 100 fs and 500 fs for both temperature and pressure, respectively.

References

(1) Handelman, A.; Kuritz, N.; Natan, A.; Rosenman, G. Reconstructive Phase Transition in Ultrashort Peptide Nanostructures and Induced Visible Photoluminescence. *Langmuir* **2016**, *32* (12), 2847–2862. https://doi.org/10.1021/acs.langmuir.5b02784.

(2) Almohammed, S.; Fedele, S.; Rodriguez, B. J.; Rice, J. H. Aligned Diphenylalanine Nanotube-Silver Nanoparticle Templates for High-Sensitivity Surface-Enhanced Raman Scattering. *J. Raman Spectrosc.* **2017**, 48.1799–1807. https://doi.org/10.1002/jrs.5254.

(3) Pei, L.; Huang, Y.; Li, C.; Zhang, Y.; Rasco, B. A.; Lai, K. Detection of Triphenylmethane Drugs in Fish Muscle by Surface-Enhanced Raman Spectroscopy Coupled with Au-Ag Core-Shell Nanoparticles. *J. Nanomater.* **2014**, *2014* (May).1–8. https://doi.org/10.1155/2014/730915.

(4) Chi, T. T. K.; Le, N. T.; Hien, B. T. T.; Trung, D. Q.; Liem, N. Q. Preparation of

SERS Substrates for the Detection of Organic Molecules at Low Concentration. *Commun. Phys.* **2017**, *26* (3).261–268. https://doi.org/10.15625/0868-3166/26/3/8053.
(5) Wu, M. C.; Lin, M. P.; Chen, S. W.; Lee, P. H.; Li, J. H.; Su, W. F. Surface-Enhanced Raman Scattering Substrate Based on a Ag Coated Monolayer Array of SiO_2 Spheres for Organic Dye Detection. *RSC Adv.* **2014**, *4* (20), 10043–10050. https://doi.org/10.1039/c3ra45255g.
(6) Görbitz, C. H. The Structure of Nanotubes Formed by Diphenylalanine, the Core Recognition Motif of Alzheimer's β-Amyloid Polypeptide. *Chem. Commun.* **2006**, 22, 2332–2334. https://doi.org/10.1111/j.1745-4514.2007.00117.x.
(7) Akdim, B.; Pachter, R.; Naik, R. R. Self-Assembled Peptide Nanotubes as Electronic Materials: An Evaluation from First-Principles Calculations. *Appl. Phys. Lett.* **2015**, *106* (18), 1–5. https://doi.org/10.1063/1.4921012
(8) Zelenovskiy, P. S.; Nuraeva, A. S.; Kopyl, S.; Arkhipov, S. G.; Vasilev, S. G.; Bystrov, V. S.; Gruzdev, D. A.; Waliczek, M.; Svitlyk, V.; Shur, V. Y.; et al. Chirality-Dependent Growth of Self-Assembled Diphenylalanine Microtubes. *Cryst. Growth Des.* **2019**, *19* (11), 6414–6421https://doi.org/10.1021/acs.cgd.9b00884.
(9) Cong, S.; Yuan, Y.; Chen, Z.; Hou, J.; Yang, M.; Su, Y.; Zhang, Y.; Li, L.; Li, Q.; Geng, F.; et al. Noble Metal-Comparable SERS Enhancement from Semiconducting Metal Oxides by Making Oxygen Vacancies. *Nat. Commun.* **2015**, *6*, 7800. https://doi.org/10.1038/ncomms8800.
(10) Zheng, Z.; Cong, S.; Gong, W.; Xuan, J.; Li, G.; Lu, W.; Geng, F.; Zhao, Z. Semiconductor SERS Enhancement Enabled by Oxygen Incorporation. *Nat. Commun.* **2017**, *8* (1), 1–11. https://doi.org/10.1038/s41467-017-02166-z.
(11) Smidstrup, S.; Markussen, T.; Vancraeyveld, P.; Wellendorff, J.; Schneider, J.; Gunst, T.; Verstichel, B.; Stradi, D.; Khomyakov, P. A.; Vej-Hansen, U. G.; et al. QuantumATK: An Integrated Platform of Electronic and Atomic-Scale Modelling Tools. *J. Phys. Condens. Matter* **2020**, *32* (1), 1–39. https://doi.org/10.1088/1361-648X/ab4007.
(12) Perdew, J. P.; Burke, K.; Ernzerhof, M. Generalized Gradient Approximation Made Simple (Vol 77, Pg 3865, 1996). *Phys. Rev. Lett.* **1997**, *78* (7), 1396–1396.
(13) van Setten, M. J.; Giantomassi, M.; Bousquet, E.; Verstraete, M. J.; Hamann, D. R.; Gonze, X.; Rignanese, G. M. The PSEUDODOJO: Training and Grading a 85 Element Optimized Norm-Conserving Pseudopotential Table. *Comput. Phys. Commun.* **2018**, *226*, 39–54. https://doi.org/10.1016/j.cpc.2018.01.012.
(14) Ferreira, L. G.; Marques, M.; Teles, L. K. Approximation to Density Functional Theory for the Calculation of Band Gaps of Semiconductors. *Phys. Rev. B - Condens. Matter Mater. Phys.* **2008**, *78* (12), 1–9. https://doi.org/10.1103/PhysRevB.78.125116.
(15) Kanoun, M. B.; Goumri-Said, S.; Schwingenschlögl, U.; Manchon, A. Magnetism in Sc-Doped ZnO with Zinc Vacancies: A Hybrid Density Functional and GGA + U Approaches. *Chem. Phys. Lett.* **2012**, *532*, 96–99. https://doi.org/10.1016/j.cplett.2012.02.055.
